# Increased extreme hourly precipitation over China’s rice paddies from 1961 to 2012

**DOI:** 10.1038/s41598-020-67429-0

**Published:** 2020-06-30

**Authors:** Yiwei Jian, Jin Fu, Bengang Li, Feng Zhou

**Affiliations:** Laboratory for Earth Surface Processes, Sino-France Institute of Earth Systems Science, College of Urban and Environmental Sciences, Peking University, Beijing, 100871 People’s Republic of China

**Keywords:** Climate change, Climate-change impacts, Environmental sciences

## Abstract

Rice yield have been affected by the increased extreme precipitation events in recent decades. Yet, the spatio-temporal patterns of extreme precipitation by rice type and phenology remain elusive. Here, we investigate the characteristics of four extreme precipitation indices across China’s rice paddy and their potential association with crop yields, by using hourly precipitation data from 1,215 stations and rice phenology observations from 45 sub-regions. The data indicate that hourly extreme precipitation have significantly increased in 1961–2012 for single rice and early rice in China but not for late rice. Rice were mainly exposed to extreme precipitation from transplantation to flowering stages. The frequency and proportion of extreme precipitation were significantly increased by 2.0–4.7% and 2.3–2.9% per decade, respectively, mainly in south China and Yangtze River Basin. The precipitation intensity and maximum hourly precipitation were increased by 0.7–1.1% and 0.9–2.8% per decade, respectively, mainly in central China and southeast coastal area. These extreme precipitation indices played a role as important as accumulated precipitation and mean temperature on the interannual variability of rice yields, regardless of rice types. Our results also highlight the urgencies to uncover the underlying mechanisms of extreme precipitation on rice growth, which in turn strengthens the predictability of crop models.

## Introduction

China, the biggest rice producer around the world, has a total rice sown area of 30 million hectares at present. However, rice was experiencing frequent extreme climate events that led to a large instability in rice production^1,2^. Long-term exposure of rice growth to extreme climate has raised our concerns^3,4^, because of the importance of the global or regional food security^5^. Currently there were a few studies focusing on the spatio-temporal patterns of extreme precipitation events over rice planting areas, especially with highly diverse rice variety, planting time and phenology in China^6^.

Precipitation extremes have been increasing globally in frequency, intensity and extent over the past decades^7–9^. The maximum daily precipitation, maximum consecutive 5-day precipitation and total precipitation from days > 95th percentile have increased by 5%, 4% and 20% during the period 1900–2010, respectively^10^. However, conclusions varied with regions and seasons^11,12^. For example, the proportion and frequency of extreme precipitation significantly increased in Eastern China while decreased in North China over the past five decades due to heterogeneous terrain and climate conditions^13,14^. By analyzing 265 stations in South Asia in 1961–2000, an increasing trend of extreme precipitation was identified in tropical regions, while decreasing in the Himalaya and desert regions^15^. Significant increase in maximum daily precipitation were found from June to August, but no obvious elevation or even decreasing during the rest of the year^16^.

Other than the possible disasters like flash floods and mudslides, extreme precipitation events have different influences on crop growth (or yield) when the events occurred in critical growing periods^6,17,18^. A positive correlation was found between rice yield and extreme precipitation events in India^19^, while it was negative in the Philippines^20^. Specifically, extreme precipitation slightly influenced rice growth at its tillering stage, while it is unfavorable for rice pollination at early rice flowering stage^21^. Long spells of rainfall at the ripening stage resulted in yield reduction due to lodging and waterlogging and impeded mechanical harvesting^22^. Moreover, extreme precipitation influenced crop yield through physiochemical and physiological mechanisms. Photosynthetic rate was increased by extreme precipitation by adjusting the stomatal opening of leaf surface of bean and pea^23^, while it was decreased due to large nutrient loss of leaf epidermis of peatland^24^. Thus, it is necessary to identify extreme precipitation indices during different rice-growing periods and unravel the potential effects of extreme precipitation on rice growth.

Most of previous studies were conducted based on daily datasets. However, extreme precipitation events often occurred in a short time from less than 1 h to a few hours^25^. Furthermore, trend analyses using hourly data could better reveal the temporal dynamics at the local scale and retain more information about precipitation pattern, especially in regions with substantial seasonal variability and undulating topography^11^. For instance, Prein et al.^26^ found that the hourly extreme precipitation increased in majority of the United States from 2001 to 2013 and expected to increase along with global warming in the future. Li et al.^27^ estimated the threshold values of hourly rainfall intensity for a 5-year return period and revealed significant regional differences over eastern China. Luo et al. ^28^ investigated synoptic situations of extreme hourly precipitation over China and suggested complicated regional features in the occurrence frequency and intensity of precipitation extremes.

The objective of this study is to provide an insight into extreme precipitation indices over rice paddy fields, including the frequency, intensity, proportion and maximum hourly precipitation (see “Methods” section). Using hourly precipitation data (1,215 stations) and rice phenology observations (45 sub-regions), we investigated the spatiotemporal patterns of extreme precipitation indices over three rice cropping systems (i.e., single rice, early rice and late rice) during 1961–2012, and tested the association between the long-term exposure to extreme precipitation and rice yield in the last three decades (1981–2012) due to data limitation (see “Methods” section). In addition, we discussed about the long-term goals in quantifying risks of extreme precipitation on rice production and associated model improvements, as well as policy implications to mitigate the losses from extreme precipitation events.

## Results

### Extreme precipitation indices by crop and phenology

**Figure 1:**
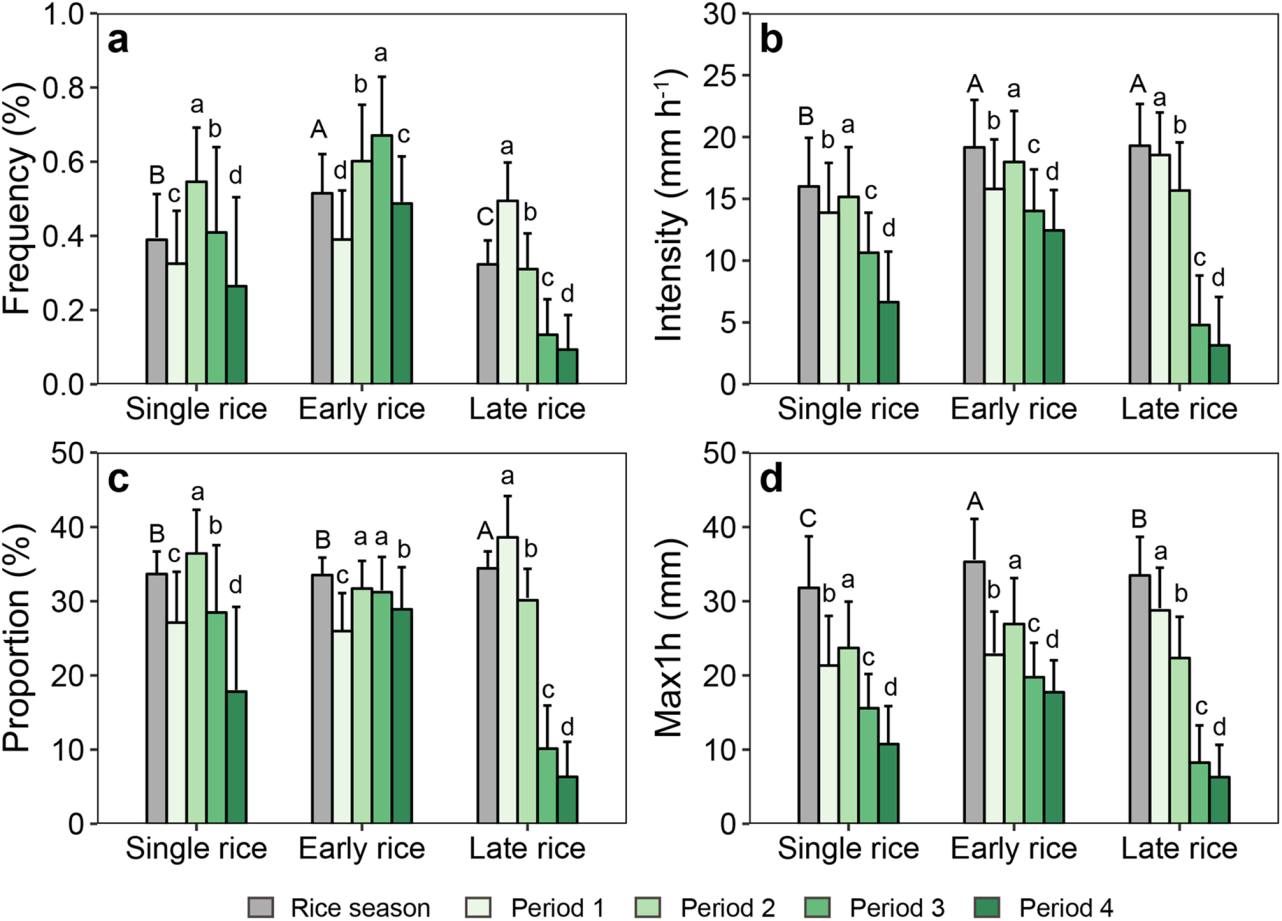
Extreme precipitation indices by rice type and growth period in 1961–2012. (**a**) Frequency of extreme precipitation; (**b**) intensity of extreme precipitation; (**c**) proportion of extreme precipitation; (**d**) maximum hourly precipitation, note that the value during whole rice growing season differs with the maximum of those in 4 growth periods because the time of maximum precipitation event differs by year and site. Period 1 represents the stage from transplanting to tillering, period 2 for the stage from the end of tillering to the end of flowering, period 3 for the stage from the end of flowering to doughty, and period 4 for the stage from maturity to harvesting. Error bar indicates one standard deviation of extreme precipitation indices due to spatial variation. Different letters indicate there were significant differences between growing periods at the 5% level. Figures were generated in R version 3.6.0 (www.r-project.org)^64^.

The extreme precipitation varied greatly among different rice growing periods, but not among three rice types (Fig. 1). For the entire rice growing season, the frequency of extreme precipitation of early rice (0.51 ± 0.11%) was significantly higher than that of single rice (0.39 ± 0.12%, p < 0.001) and late rice (0.32 ± 0.06%, p < 0.001) (Fig. 1a). In addition, the frequency in the period 2 and 3 were significant higher than those in period 1 and 4 for single rice and early rice (p < 0.001), while for late rice higher frequency occurred in period 1 and 2 (Fig. 1a). The intensity and maximum hourly precipitation differed significantly by growth period (p < 0.001) and were higher in the first two periods for each rice type (Fig. 1b,d). There were no significant differences of the proportion of extreme precipitation (range within 33.5–34.4%) between cropping systems for the entire rice season, and significant higher proportion of extreme precipitation was found for periods 2 and 3 for single rice and early rice (Fig. 1c).

**Figure 2:**
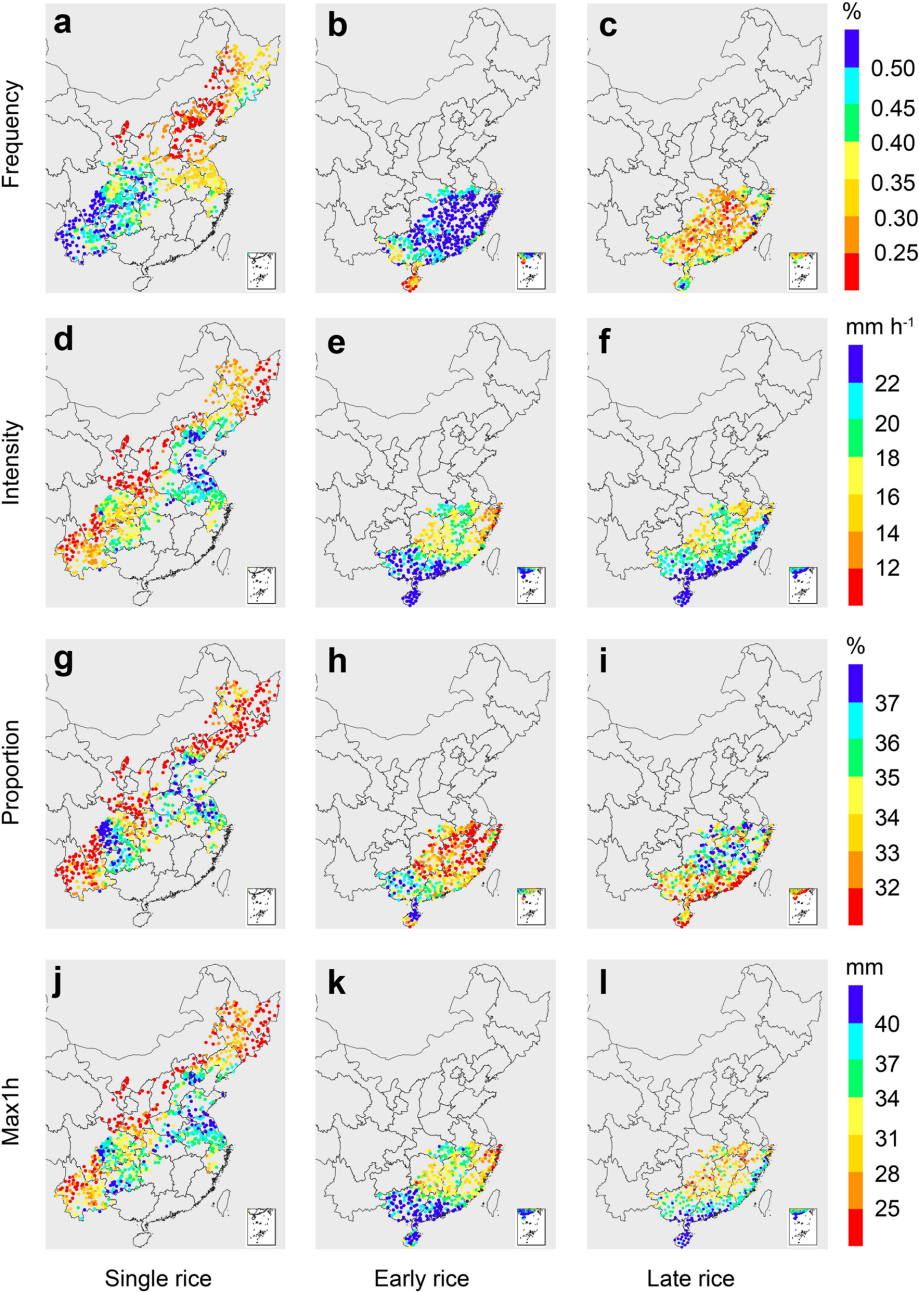
Patterns of extreme precipitation indices for rice growing season averaged over the period 1961–2012. (**a**–**c**) Frequency of extreme precipitation; (**d**–**f**) intensity of extreme precipitation; (**g**–**i**) proportion of extreme precipitation; (**j**–**l**) maximum hourly precipitation for single rice region (left) , early rice region (middle) , late rice region (right) during 1961–2012. Maps were generated in R version 3.6.0 (www.r-project.org)^64^.

Notable spatial discrepancies were detected for all four extreme precipitation indices based on the averaged data over 1961–2012 (Fig. 2). For the single rice, the frequency of extreme precipitation was relatively higher in southwest China (0.34–0.96%) than the east and the northeast (0.14–0.56%) (Fig. 2a). Intensity of the extreme precipitation was relatively lower in the southwest but reached the highest in North China Plain (Fig. 2d). Similar patterns were found for the proportion and maximum hourly precipitation (Fig. 2g,j). For early rice, the frequency of extreme precipitation was generally high in most provinces (0.39–0.72%, Fig. 2b). The intensity, proportion and maximum hourly precipitation showed similar spatial patterns with the hotspots in Hainan, Guangxi, and Guangdong (Supplementary Fig. S2a, Fig. 2e,h,k). For late rice, the frequency ranged from 0.17 to 0.58% (Fig. 2c). The intensity and maximum hourly precipitation showed similar patterns with a gradual decrease from maritime areas to the inland (Fig. 2f,l). In contrast, hotspots of the proportion were primarily distributed in Hunan, Jiangxi, and Fujian (35.7% to 40.6%), whereas low values were found in south China (28.0% to 36.1%, Fig. 2i). These proportions were smaller than previous studies that used the 90th percentile threshold^29,30^. Details of the patterns of four indices in different growth periods could be found in Supplementary Figs. S3–S6.

**Figure 3:**
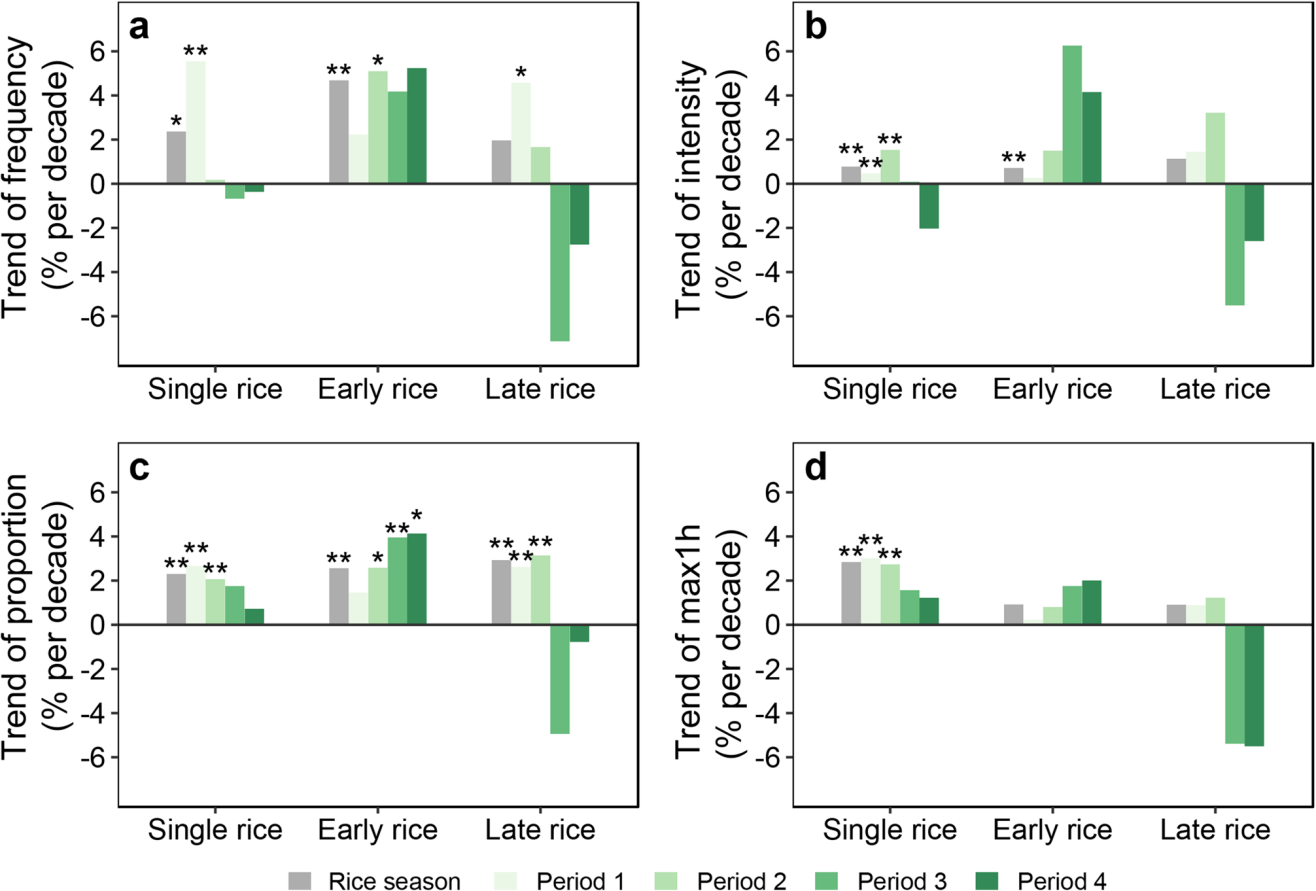
Temporal trends of extreme precipitation indices by rice type and growth period in 1961–2012. (**a**) Frequency of extreme precipitation; (**b**) intensity of extreme precipitation; (**c**) proportion of extreme precipitation; (**d**) maximum hourly precipitation, with *p < 0.05 and **p < 0.01. The definition of growth period 1 is same as Fig. 1. Figures were generated in R version 3.6.0 (www.r-project.org)^64^.

The trends of extreme precipitation indices were further detected during rice growing season over the period 1961–2012 (Fig. 3). For single rice, the indices exhibited significantly increasing trends over the entire growing season, with 2.4% per decade (p = 0.02) for frequency, 0.8% per decade (p < 0.001) for intensity, 2.3% per decade (p < 0.001) for the proportion, and 2.8% per decade (p < 0.001) for maximum hourly precipitation. Temporal trends of four indices for early rice growing season were similar to single rice, except for maximum hourly precipitation (0.9% per decade, p = 0.07). In contrast, the proportion of extreme precipitation showed a significant increment for late rice (2.9% per decade, p < 0.001), while other indices showed insignificant upward tendencies. Large tendency differences were found between the four growing periods. An increasing trend of four indices was found within periods 1 and 2 for single rice (Fig. 3). For early rice, no significant upwards tendencies were found, except the frequency in period 2 with a notable increase at rate of 5.1% per decade (p = 0.03; Fig. 3a,b,d). Trends of the proportion were shown with a significant increase in the latter three periods ranged from 2.6 to 4.1% per decade (p < 0.05; Fig. 3c). For late rice, all four indices showed upward tendencies during periods 1 and 2, of which the proportion of extreme precipitation showed a significant trend (2.6% to 3.1% per decade, p < 0.01).

**Figure 4:**
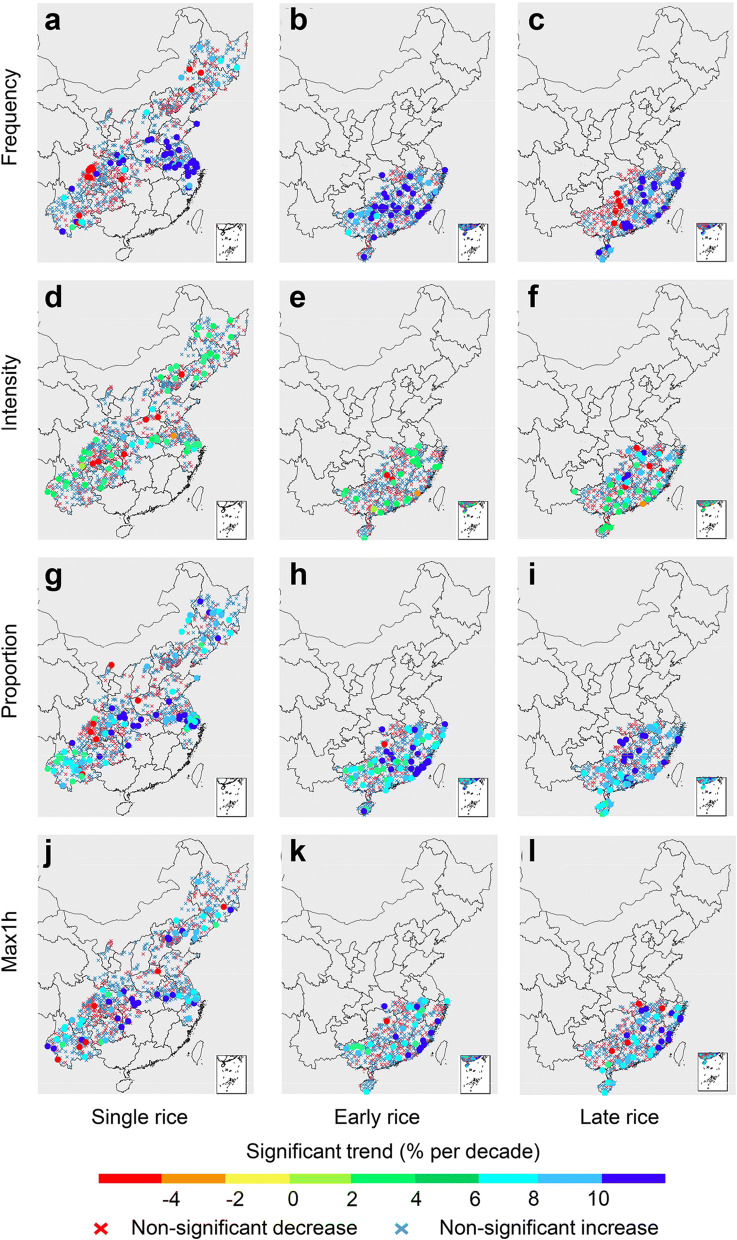
Patterns of the temporal trends of extreme precipitation indices in 1961–2012. (**a**–**c**) Frequency of extreme precipitation; (**d**–**f**) intensity of extreme precipitation; (**g**–**i**) proportion of extreme precipitation; (**j**–**l**) maximum hourly precipitation for single rice region (left), early rice region (middle), late rice region (right). Red crosses indicated for insignificant negative trends, blue crosses indicated for insignificant positive trends, while dots indicated for significant trends (p < 0.05). Maps were generated in R version 3.6.0 (www.r-project.org)^64^.

The trends of extreme precipitation indices were spatially heterogeneous (Fig. 4). For single rice, about two thirds (65%) of stations showed an increasing frequency of extreme precipitation, 6.2% of which were statistically significant (from 4.8 to 18.5% per decade; Fig. 4a). For early rice, 80% of stations showed an increasing frequency, while 11.4% was significant (from 7.1 to 33.1% per decade; Fig. 4b). More than two thirds of stations showed a relatively low increasing trend of the intensity (range from 1.9 to 13.1% per decade; Fig. 4d–f). ~ 75% of stations were shown with increasing tendencies of the proportion in three rice types, 10.6–16.3% of which were with significant increase at a rate of 3.6–22.5% per decade that were equally distributed over paddy fields (Fig. 4g–i). Trend analysis indicated a stronger changes in four growing periods over the last 50 years, except for late rice during periods 3 and 4 (Supplementary Figs. S7–S10). In summary, increasing trends for rice growing season since 1961 were found in the most of stations for the proportion of extreme precipitation (70.6–80.1%, with 10.6–16.3% significant) and for maximum hourly precipitation (67.0–69.9%, with 7.5–8.7% significant), while mixed trends were found for the frequency and intensity of extreme precipitation.

### Association between extreme precipitation and rice yield

**Figure 5:**
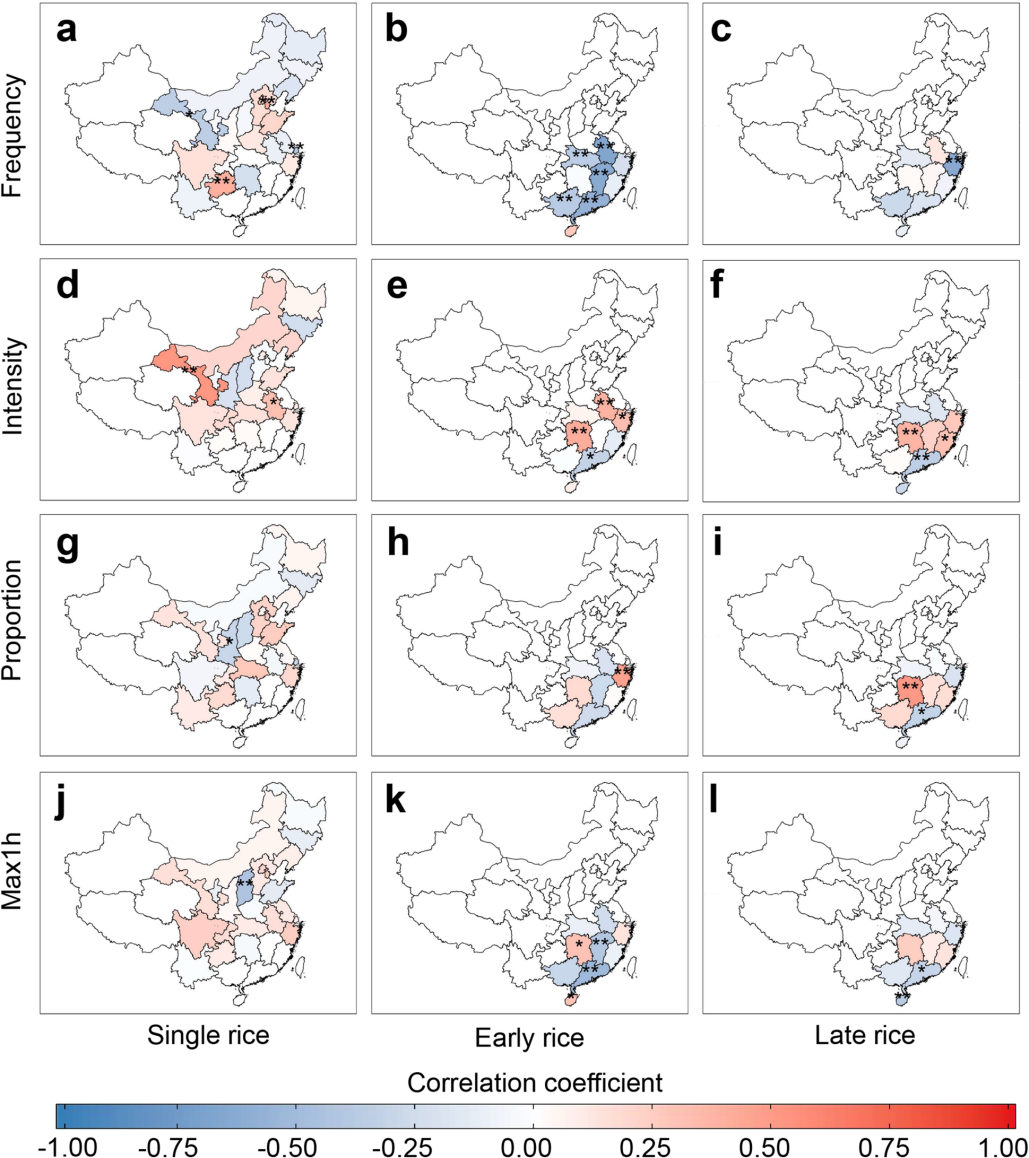
Correlation coefficient between rice yields and extreme precipitation indices in 1981–2012 at the provincial scale. (**a**–**c**) Frequency of extreme precipitation of single rice (left) , early rice (middle), and late rice (right); (**d**–**f**) intensity of extreme precipitation; (**g**–**i**) proportion of extreme precipitation; (**j**–**l**) maximum hourly precipitation. Asterisks indicate the significance of each correlation. *p < 0.1 and **p < 0.05. Maps were generated in R version 3.6.0 (www.r-project.org)^64^.

A negative correlation between the frequency and single rice yield was found in the Northern China and Eastern China, while the correlation was positive in southwest and the North China Plain (Fig. 5a). Early rice yield was significantly and negatively correlated with the frequency of extreme precipitation (Fig. 5b). Negative correlations between frequency and rice yield were also found for late rice at coastal area (Fig. 5c). The relationship between the intensity of extreme precipitation and yield of single rice showed positive correlations in most provinces (Fig. 5d). Negative correlations were further found at coastal areas of early and late rice, while provinces in the middle and lower reaches of the Yangtze river Basin, such as Hunan and Zhejiang, showed positive relationships (r = 0.33 to 0.52, p < 0.1) (Fig. 5e,f). The spatial pattern of correlations between the proportion of extreme precipitation and single rice yield was not notable (Fig. 5g). Proportion of extreme precipitation showed negative correlations with early and late rice yields at coastal area, but positive correlations in the inner regions (Fig. 5h,i). Similar spatial distributions were found for the correlation between maximum hourly precipitation and rice yield (Fig. 5j–l). Details of the patterns of the correlations in different growth periods could be found in Supplementary Tables S3–S5.

**Figure 6:**
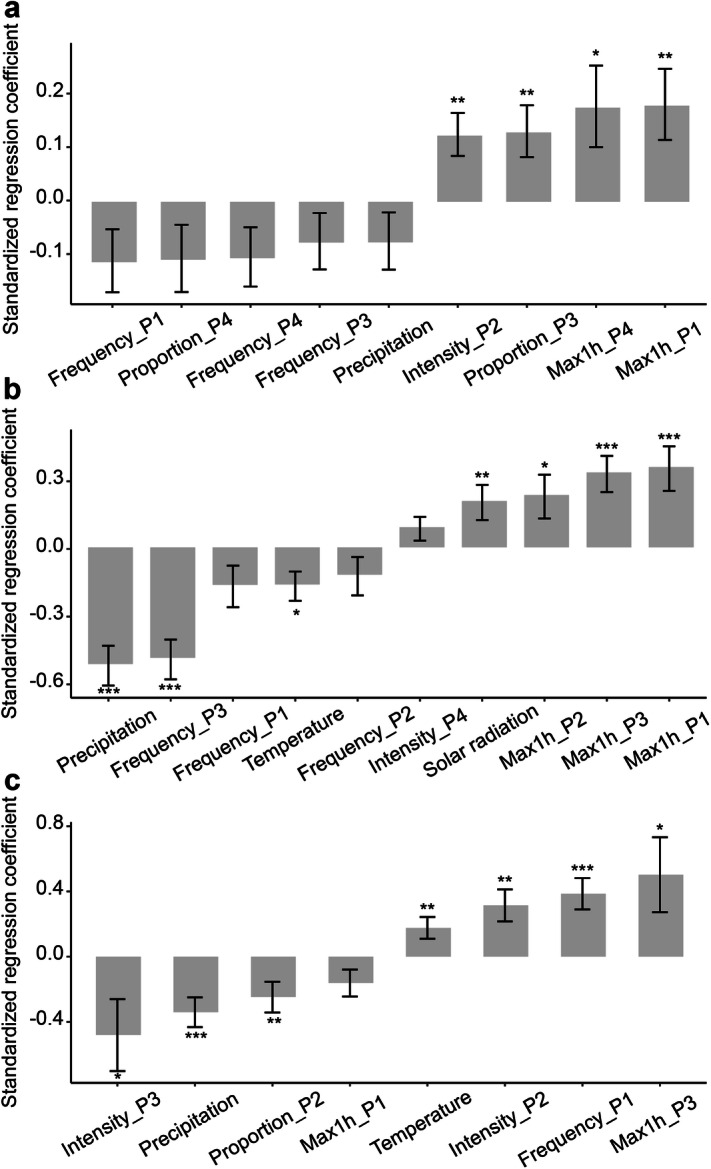
Regression coefficients (± S.E.) show the magnitude of the effect of each variable in a multiple regression. (**a**) Single rice (n = 713); (**b**) early rice (n = 279); (**c**) late rice (n = 279). Asterisks indicate the significance of each predictor. ***p < 0.001; **p < 0.01; *p < 0.05. P1 to P4 represent the four growth periods. Figures were generated in R version 3.6.0 (www.r-project.org)^64^.

We further implemented stepwise regression to identify the determinants of rice yield variability for three rice types across rice-growing provinces (Fig. 6). Climate factors explained 41% for early rice, 11% for late rice, but only 3% for single rice (Supplementary Table S6). Results highlighted that extreme precipitation were as important as accumulated precipitation and mean temperature on the inter-annual yield differences, regardless of rice types. For single rice, significantly positive effects were found for the maximum hourly precipitation in periods 1 and 4, the proportion in period 3 and the intensity of extreme precipitation in period 2 (Fig. 6a). For early rice, the frequency of extreme precipitation in period 3 has significantly negative effects on yield variability (r =  − 0.49, p < 0.001). The effects of maximum hourly precipitation in the former three periods were opposite in direction to the frequency (r = 0.23 to 0.36; Fig. 6b). For late rice, inter-annual variability of rice yield was negatively related to the intensity of extreme precipitation in period 3 (r =  − 0.48, p = 0.03) and to the proportion in period 2 (r =  − 0.25, p = 0.009). In addition, positive relationships were found for maximum hourly precipitation in period 3, the frequency in period 1, and the intensity in period 2 (Fig. 6c).

## Discussion

Our analyses based on observations from 1,215 stations revealed that hourly extreme precipitation have significantly increased in 1961–2012 for single rice and early rice in China but not for late rice. This dataset owned higher spatial and temporal resolutions than previous studies. For instance, Zhang and Zhai^31^ examined hourly precipitation trends using the data from 480 stations from May to September in 1961–2000. This study found a positive trend of the frequency (2.5–7.5% per decade) in Northeast China and the middle and lower reaches of the Yangtze river Basin, while not evident for the intensity. Li et al.^29^ analyzed the hourly precipitation data of 1,141 stations during 1982–2012, indicating a relatively low increment trend (< 1% per decade) of the frequency located in south China and Huang-Huai-Hai Plain, and even a decrease in Sichuan and northeast China. Such discrepancies indicated the effects of time period and available stations on the spatiotemporal patterns of hourly extreme precipitation. In addition, the analysis presented in this study helped describing the large variations in historical trend of extreme precipitation across rice producing regions of China.

This study is the first time to quantify the characteristics of extreme precipitation by rice growing periods. Results suggest that the rice growth are mainly exposed to extreme precipitation events at earlier stages. This is comparable to previous studies that presented the risks of extreme precipitation only at regional scale. For example, Xu et al.^32^ stated that over 60% of 52 stations in Jiangsu province showed increasing trends of the frequency and intensity of daily extreme precipitation during June–August of 1961–2012. Thus, our study based on the observations from 1,215 stations over the past five decades provides comprehensive information, which may be beneficial for farmers or policy makers to optimize their rice-cropping systems to adapt extreme precipitation.

The frequency and proportion of extreme precipitation were negatively correlated with historical yields of early or late rice, especially in southeast coastal area where were the major rice producing areas of China. Such negative correlations were also found in Hainan Island^33^, southeast China^34^ and India^35^. The findings have several policy implications for adapting extreme precipitation events. First, optimizing farm management measures (e.g. shallow-wet irrigation, reseeding and fixing) and investigating in the drainage facilities, including canals, ponds and pump equipment, can improve drainage efficiencies and farmer’s adaptive capacity^36^. Second, public services, such as providing disaster warning information and technical guidance, are benefit for farmers' prevention awareness and access to advanced technologies^37^. Further, breeding rice that carry a diversity of resistance genes to environmental stress seem to be fundamental but with great challenges for agricultural sustainability in China^2^.

However, there are still certain limitations within our analyses. First, the high-resolution (1 × 1 km) maps of rice paddy were helpful to filter meteorological stations close to the small paddy fields across China, but only reflected spatial distributions from 1990 to 2010. We then compared the meteorological stations selected by two additional union sets of rice paddy layers: one was from the History Database of the Global Environment (HYDE 3.2.1)^38^ at 5-arc-min scale, the other one was from the high-resolution maps which were used in this study but resampled into a 5-arc-minute grid dataset. Supplementary Figure S11 indicate that ~ 20% of meteorological stations were not overlapped, mainly in southwest China and the North China Plain. This result implied that the assumption that the pattern remained unchanged before 1990 may introduce additional uncertainties^39,40^. Second, the number of meteorological stations is 40% less before 1980, which would distort the regionally averaged trend analyses^41^. Sensitivity analysis was then conducted to determine the consistency of regional trends using different group of stations. The regional trends of extreme precipitation indices were insensitive to the number of meteorological stations, except for the intensity of extreme precipitation that showed opposite trends between two different groups of station (294 v.s. 803 for single rice, 220 v.s. 412 for double rice, Supplementary Fig. S12). Third, the 95th percentile thresholds were defined using the records in 1981–2010, because of the maximum number of stations meeting the strict data availability requirements. We thus analysed the sensitivity of the 95th percentile thresholds to different base periods (1981–2010, 1976–2005, 1971–2000, 1971–2010, and 1961–2012). Fortunately, Supplementary Table S7 indicated a small differences (< 4%) in the 95th percentile thresholds between them. Last, the spatiotemporal pattern of extreme precipitation indices may be sensitive to the length of time periods. Additional analyses in Supplementary Figs. S13–S16 indicated an obvious difference in the temporal trend of extreme precipitation indices between the whole time period (1961–2012) and shorter periods (1990–2010 and 1980–2010). However, there are no obvious discrepancies in the mean values or spatial patterns.

In addition, our study only focused on the association of crop growth in response to extreme precipitation, but the underlying mechanisms remains elusive. This still limits our capabilities of using land surface models to simulate the response of crop phenology or morphology to extreme precipitation events^42,43^. At present, most of land surface models considered the precipitation as a factor to regulate soil water content^44,45^ and nutrient losses via runoff and leaching^46^, but neglected the physical and physiological effects of extreme precipitation on crop growth^17,18,47,48^. In addition, the models are unable to reflect the spatial and temporal variations of the response of crop growth to extreme precipitation. Therefore, the long-term goal is to uncover the mechanisms and quantify the risks of extreme precipitation on rice growth, which in turn strengthens the predictability of the models in response to extreme precipitation.

## Methods

### Definitions

Four extreme precipitation indices were calculated not only in the entire rice-growing season but also for each growing period. The 95th percentile was selected as a threshold to represent extreme precipitation, which was recommended by the ETCCDI^49^. All hourly precipitation above 0.1 mm occurring throughout the base period (1981–2010) were sorted in ascending order at each station to determine the threshold^50,51^. The base period was chosen based on the rules of maximizing the number of stations meeting the strict data availability requirements^52^. Four extreme precipitation indices were used to characterize extreme hourly precipitation during rice growing season (Supplementary Table S1), including three percentile-based indices (the frequency, intensity and proportion of extreme hourly precipitation) and one absolute indices (maximum hourly precipitation, i.e., max 1 h). The frequency was defined as the fraction of the number of hours when hourly precipitation exceeded the 95th percentile threshold to the length of the growth period in hours. The intensity was calculated as the mean of extreme hourly precipitation. The proportion was defined as the ratio of the amount of extreme precipitation to total precipitation amount. Further details of these indices are shown in Supplementary Table S1.

### Datasets

Hourly precipitation dataset was obtained from the National Meteorological Information Center of the China Meteorological Administration (https://data.cma.cn/en). Observations were collected from 2,420 nationally distributed meteorological stations during the period 1961–2012. Precipitation was automatically measured by either tipping-bucket or self-recording siphon rain gauge, with strict quality assurance including the climatological limit value test, the time consistency check, and the internal consistency check^28,29^. An entire year of observations would be removed from the dataset if there were more than 2% of hourly observations missing in that year. Two types of stations were also excluded from our analysis: (i) stations with the observation period less than 30 years, and (ii) stations with inconsecutive observations in more than 10% of the observation year. After that, missing values were still around 0.2% of the total population, which have little impact on our results.

The hourly precipitation dataset was further filtered only for the rice-growing season across China’s rice paddies. First, a gridded rice paddy map at the spatial resolution of 1 × 1 km was developed as the union set of land use layers derived from the Landsat during the period 1990–2010^53^, where assumed the pattern remained unchanged before 1990. Second, the meteorological stations were selected if there were rice paddies located within a buffer zone within 20 km in radius. The final dataset contains the hourly precipitation observations from 1,215 stations (i.e., 813 for single rice and 412 for double rice; Supplementary Fig. S1). The selected stations were evenly distributed across China’s rice paddies that well represented the contrasting environmental conditions compared to the selections using larger or smaller buffer zones (i.e., the radius of 10 km, 30 km, and 50 km). Third, the hourly precipitation observations were extracted from the period from rice transplanting to harvesting stage. Phenological information for single and double rice were retrieved from the agro-meteorological field observation network^54,55^, including the period from transplanting to tillering (period 1), the period from the end of tillering to the end of flowering (period 2), the period from the end of flowering to doughty (period 3), and the period from maturity to harvesting (period 4) in each of 45 rice-cropping sub-regions (Supplementary Figs. S1, S2 and Supplementary Table S2). Recent studies suggest that the length of rice growing period hardly varied, i.e. on average 1.0, 0.2 and 2.0 day per decade increase during 1991–2012 for early, late and single rice, respectively^54^. Therefore, rice phenology was kept constant throughout all the observation periods in this study.

### Statistical analysis

To determine the significance of extreme precipitation indices between rice types and rice growing periods, homogeneity tests were carried out. According to the results of Shapiro–Wilk test, extreme precipitation indices are not normally distributed. Therefore, the difference of extreme precipitation indices between single rice and double rice was tested for the entire rice season using nonparametric Mann–Whitney U test. Wilcoxon signed-rank test, which is suitable for paired sample, was used to test the differences between early rice and late rice for the entire rice season, and between four rice growing periods.

Temporal trends were examined for extreme precipitation indices for the 1,215 stations during each rice-growing period from 1961 to 2012, and for each rice type. Trend detection were carried out by Mann–Kendall nonparametric test (M–K test)^7,56^. A positive Z value indicates an increasing trend while a negative Z value indicates a decreasing trend. The statistical significance was assessed at the 5% level. In addition, Sen’s slope estimator was applied to quantify the trend of extreme precipitation indices during 1961–2012. The slope is the median among all combination of calculations. The M–K test was based on the assumption that the time series was independent since serial correlation could lead to unreliable statistical significance of trend^57,58^. Therefore, autocorrelation test for each extreme precipitation index at each station was performed before applying the M–K test. The lag-1 serial autocorrelation coefficients were not significant, suggesting that the time series were independent and the following trend analysis could be applied to the original values of time series.

We conducted the correlation analysis between rice yield and extreme precipitation during rice growing periods. Rice yield data were obtained at the provincial level during the period 1981–2012 from the National Bureau of Statistics (https://www.stats.gov.cn/english/). Four extreme precipitation indices were determined as area-weighted average value when being aggregated from sites into provincial level. The corresponding area for each station was determined based on Thiessen polygon. Prior to correlation analysis, we used the first-order difference method to detrend both crop yields and extreme precipitation indices^59^. This method can avoid the effects due to non-climatic factors (e.g., technology and management improvements). Spearman rank correlation coefficients were then calculated by rice type and province.

We further conducted multiple linear regression models for each of three rice types to test whether rice yield variability depended on extreme precipitation indices across provinces and time periods. In addition to extreme precipitation, each regression model included growing-season accumulated precipitation, mean temperature and mean solar radiation that were considered as effective variables explaining crop yield variability in previous studies^60–62^. It should be noted that extreme precipitation indices by growth period were initially considered in regression models^63^. These three predictor variables were extracted from the China Meteorological Forcing Dataset (https://doi.org/10.6084/m9.figshare.c.4557599.v1), but further aggregated as area-weighted average values at the provincial scale. For each regression model, we filtered the predictor variables to avoid collinearity between them using Variance Inflation Factors (VIFs) and minimizing the number of variables until all remaining variables fell within the predetermined threshold (i.e., VIF < 5). Because predictors included in the models were measured in different units and have various ranges of values, we standardized each of them, across all provinces and over the full time period, to have zero mean but its own unique variance. Such standardization performed before analyses, and enabled quantitative comparisons of the resulting model coefficients for the predictor variables. To avoid over-fitting, each regression model was then simplified using the Akaike Information Criterion (AIC) values by the implementation of stepwise regression.

## Supplementary information

Supplementary information

## Data Availability

All data used in figure creation are publicly available online at https://figshare.com/articles/Extreme_precipitation_Dataset_of_China/12115563.

## References

[1] Iizumi T, Ramankutty N (2016). Changes in yield variability of major crops for 1981–2010 explained by climate change. Environ. Res. Lett..

[2] Bailey-Serres J, Parker JE, Ainsworth EA, Oldroyd GED, Schroeder JI (2019). Genetic strategies for improving crop yields. Nature.

[3] Tubiello FN, Soussana JF, Howden SM (2007). Crop and pasture response to climate change. Proc. Natl. Acad. Sci. USA.

[4] Lesk C, Rowhani P, Ramankutty N (2016). Influence of extreme weather disasters on global crop production. Nature.

[5] Challinor AJ (2014). A meta-analysis of crop yield under climate change and adaptation. Nat Clim Change.

[6] Zhu X, Troy TJ (2018). Agriculturally relevant climate extremes and their trends in the world's major growing regions. Earth's Fut..

[7] Alexander LV (2006). Global observed changes in daily climate extremes of temperature and precipitation. J. Geophys. Res..

[8] Sillmann J, Kharin VV, Zwiers FW, Zhang X, Bronaugh D (2013). Climate extremes indices in the CMIP5 multimodel ensemble: Part 2. Future climate projections. J. Geophys. Res.-Atmos..

[9] Myhre G (2019). Frequency of extreme precipitation increases extensively with event rareness under global warming. Sci. Rep..

[10] Donat MG (2013). Updated analyses of temperature and precipitation extreme indices since the beginning of the twentieth century: The HadEX2 dataset. J. Geophys. Res. Atmos..

[11] Barbero R, Fowler HJ, Lenderink G, Blenkinsop S (2017). Is the intensification of precipitation extremes with global warming better detected at hourly than daily resolutions?. Geophys. Res. Lett..

[12] Tong S (2019). Spatial and temporal variability in extreme temperature and precipitation events in Inner Mongolia (China) during 1960–2017. Sci. Total Environ..

[13] Miao C, Sun Q, Borthwick AG, Duan Q (2016). Linkage between hourly precipitation events and atmospheric temperature changes over china during the warm season. Sci. Rep..

[14] Miao CY, Duan QY, Sun QH, Lei XH, Li H (2019). Non-uniform changes in different categories of precipitation intensity across China and the associated large-scale circulations. Environ. Res. Lett..

[15] Sheikh MM (2015). Trends in extreme daily rainfall and temperature indices over South Asia. Int. J. Climatol..

[16] Cheong WK (2018). Observed and modelled temperature and precipitation extremes over Southeast Asia from 1972 to 2010. Int. J. Climatol..

[17] Li Y, Guan K, Schnitkey GD, DeLucia E, Peng B (2019). Excessive rainfall leads to maize yield loss of a comparable magnitude to extreme drought in the United States. Glob. Change Biol..

[18] Rosenzweig C, Tubiello FN, Goldberg R, Mills E, Bloomfield J (2002). Increased crop damage in the US from excess precipitation under climate change. Glob. Environ. Change.

[19] Revadekar JV, Preethi B (2012). Statistical analysis of the relationship between summer monsoon precipitation extremes and foodgrain yield over India. Int. J. Climatol..

[20] Blanc E, Strobl E (2016). Assessing the impact of typhoons on rice production in the Philippines. J. Appl. Meteorol. Climatol..

[21] Yang L, Qin Z, Tu L (2015). Responses of rice yields in different rice-cropping systems to climate variables in the middle and lower reaches of the Yangtze River, China. Food Security.

[22] Lee MS (2013). How do extreme wet events affect rice quality in a changing climate?. Agric. Ecosyst. Environ..

[23] Hanba YT, Moriya A, Kimura K (2004). Effect of leaf surface wetness and wettability on photosynthesis in bean and pea. Plant Cell Environ..

[24] Nijp JJ (2015). Rain events decrease boreal peatland net CO2 uptake through reduced light availability. Glob. Change Biol..

[25] Barbero R (2019). A synthesis of hourly and daily precipitation extremes in different climatic regions. Weather Clim. Extrem..

[26] Prein AF (2017). The future intensification of hourly precipitation extremes. Nat. Clim. Change.

[27] Li J, Yu R, Sun W (2014). Duration and seasonality of hourly extreme rainfall in the central eastern China. Acta Meteorol. Sin..

[28] Luo Y, Wu M, Ren F, Li J, Wong W-K (2016). Synoptic situations of extreme hourly precipitation over China. J. Clim..

[29] Li D (2016). Spatiotemporal characteristics of hourly precipitation over central eastern China during the warm season of 1982–2012. Int. J. Climatol..

[30] Fu S (2016). A 31-year trend of the hourly precipitation over South China and the underlying mechanisms. Atmos. Sci. Lett..

[31] Zhang H, Zhai P (2011). Temporal and spatial characteristics of extreme hourly precipitation over eastern China in the warm season. Adv. Atmos. Sci..

[32] Xu Y, Sun L, Huang J, Wu Q, Zhu Y (2017). Spatiotemporal variation of extreme precipitation in Jiangsu Province in the past 50 years and its effect on yield of single rice. Guangdong Agric. Sci..

[33] Li M, Luo W, Li H, Liu E, Li Y (2017). Daily extreme precipitation indices and their impacts on rice yield—A case study over the tropical island in China. Theor. Appl. Climatol..

[34] Tao F, Zhang Z, Zhang S, Zhu Z, Shi W (2012). Response of crop yields to climate trends since 1980 in China. Clim. Res..

[35] Auffhammer M, Ramanathan V, Vincent JR (2012). Climate change, the monsoon, and rice yield in India. Clim. Change.

[36] Zhuang Y (2019). Effects and potential of water-saving irrigation for rice production in China. Agric. Water Manage..

[37] Huang J, Wang Y, Wang J (2015). Farmers' adaptation to extreme weather events through farm management and its impacts on the mean and risk of rice yield in China. Am. J. Agric. Econ..

[38] Klein Goldewijk, C.G.M. (Utrecht University). Anthropogenic land-use estimates for the Holocene; HYDE 3.2. DANS. 10.17026/dans-25g-gez3. (2017).

[39] Liu Z (2013). Change analysis of rice area and production in China during the past three decades. J. Geogr. Sci..

[40] Li Z (2015). Chinese rice production area adaptations to climate changes, 1949–2010. Environ. Sci. Technol..

[41] Kamiguchi K (2010). Development of APHRO_JP, the first Japanese high-resolution daily precipitation product for more than 100 years. Hydrol. Res. Lett..

[42] Gellesch E, Khan MASA, Kreyling J, Jentsch A, Beierkuhnlein C (2017). Grassland experiments under climatic extremes: Reproductive fitness versus biomass. Environ. Exp. Bot..

[43] Feng PY (2018). Impacts of rainfall extremes on wheat yield in semi-arid cropping systems in eastern Australia. Clim. Change.

[44] Moriondo M, Giannakopoulos C, Bindi M (2011). Climate change impact assessment: the role of climate extremes in crop yield simulation. Clim. Change.

[45] van der Velde M, Tubiello FN, Vrieling A, Bouraoui F (2011). Impacts of extreme weather on wheat and maize in France: Evaluating regional crop simulations against observed data. Clim. Change.

[46] Muller C (2017). Global gridded crop model evaluation: Benchmarking, skills, deficiencies and implications. Geosci. Model. Dev..

[47] Wang R, Bowling LC, Cherkauer KA (2016). Estimation of the effects of climate variability on crop yield in the Midwest USA. Agric. For. Meteorol..

[48] Iizumi T (2017). Responses of crop yield growth to global temperature and socioeconomic changes. Sci. Rep..

[49] Zhang X (2011). Indices for monitoring changes in extremes based on daily temperature and precipitation data. Wiley Interdiscipl. Rev. Clim. Change.

[50] Zhai P, Zhang X, Wan H, Pan XH (2005). Trends in total precipitation and frequency of daily precipitation extremes over China. J. Clim..

[51] Syafrina AH, Zalina MD, Juneng L (2014). Historical trend of hourly extreme rainfall in Peninsular Malaysia. Theor. Appl. Climatol..

[52] Aguilar E (2005). Changes in precipitation and temperature extremes in Central America and northern South America, 1961–2003. J. Geophys. Res..

[53] Liu J (2014). Spatiotemporal characteristics, patterns, and causes of land-use changes in China since the late 1980s. J. Geogr. Sci..

[54] Wang X (2017). Management outweighs climate change on affecting length of rice growing period for early rice and single rice in China during 1991–2012. Agric. For. Meteorol..

[55] Liao X (2010). Regional Target Production of Rice Cropping Region and Technical Specification.

[56] Westra S, Alexander LV, Zwiers FW (2013). Global increasing trends in annual maximum daily precipitation. J. Clim..

[57] Sun WY (2016). Changes in extreme temperature and precipitation events in the Loess Plateau (China) during 1960–2013 under global warming. Atmos. Res..

[58] Bayazit M, Önöz B (2007). To prewhiten or not to prewhiten in trend analysis?. Hydrol. Sci. J..

[59] Lobell DB, Field CB (2007). Global scale climate–crop yield relationships and the impacts of recent warming. Environ. Res. Lett..

[60] Roberts MJ, Braun NO, Sinclair TR, Lobell DB, Schlenker W (2017). Comparing and combining process-based crop models and statistical models with some implications for climate change. Environ. Res. Lett..

[61] Parkes B (2019). Weather dataset choice introduces uncertainty to estimates of crop yield responses to climate variability and change. Environ. Res. Lett..

[62] Yin XG, Olesen JE, Wang M, Öztürk I, Chen F (2016). Climate effects on crop yields in the Northeast Farming Region of China during 1961–2010. J. Agric. Sci..

[63] Prabnakorn S, Maskey S, Suryadi FX, de Fraiture C (2018). Rice yield in response to climate trends and drought index in the Mun River Basin, Thailand. Sci. Total Environ..

[64] Team R Core: A language and environment for statistical computing. (2018).

