# Supplementary material for: Increased extreme hourly precipitation over China’s rice paddies from 1961 to 2012
Source: Sci Rep. 2020 Jun 30;10:10609. doi: 10.1038/s41598-020-67429-0 (PMC7326977; doi:10.1038/s41598-020-67429-0)
Supplement: Supplementary file 1 — Supplementary information [file 41598_2020_67429_MOESM1_ESM.pdf]

SUPPLEMENTARY MATERIAL

Increased extreme hourly precipitation over China's rice paddies from 1961 to 2012

Yiwei Jian[1,#], Jin Fu[1,#], Bengang Li[1], Feng Zhou[1,*]

[1] Sino-France Institute of Earth Systems Science, Laboratory for Earth Surface Processes, College of Urban and Environmental Sciences, Peking University, Beijing 100871, P.R. China

# Y.W.J. and J.F. contributed equally to this work.

* Corresponding author: Phone / fax: +86 10 62756511; Email: zhouf@pku.edu.cn

Content: 16 figures and 7 tables

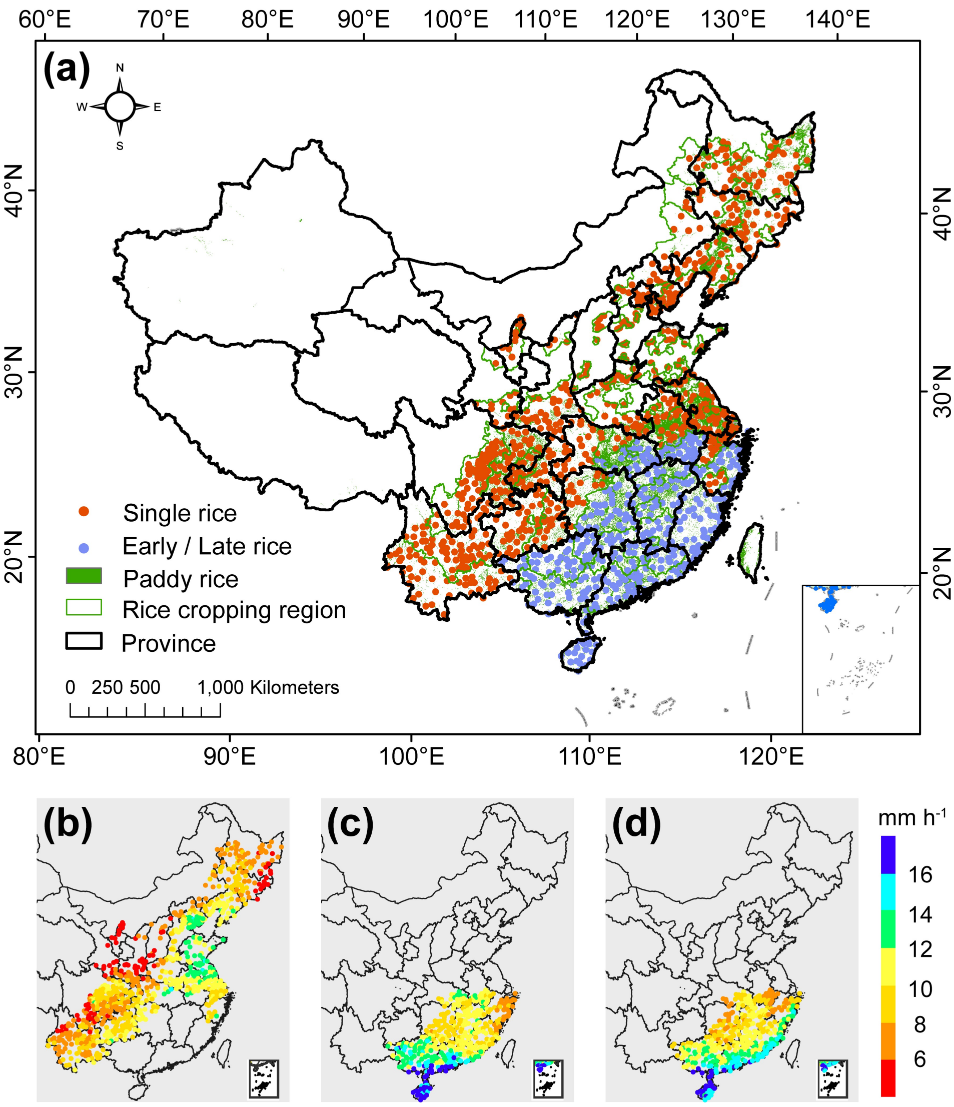

Fig. S1. Spatial distribution of (a) meteorological stations used in this study and 95th percentile threshold of extreme precipitation across paddy fields for (b) single rice, (c) early rice, (d) late rice. Note that rice cropping regions were divided by rice phenology and type. Maps were generated in R version 3.6.0 (www.r-project.org)[1].

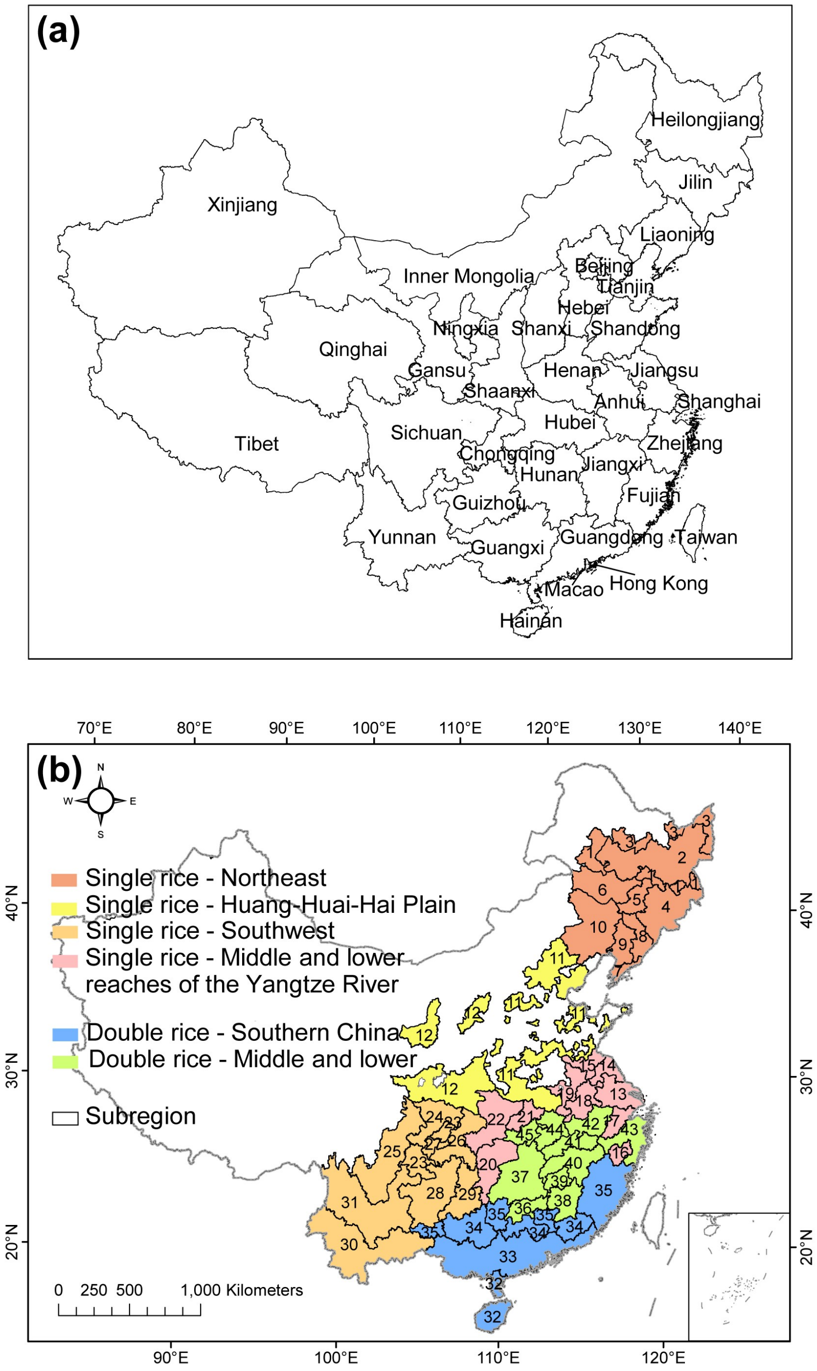

Figure S2. Locations of (a) each province and (b) rice cropping regions of China. Numbers of (b) show the sub-region of rice planting, with detailed information illustrated in Table S2. Maps were generated in R version 3.6.0 (www.r-project.org)[1].

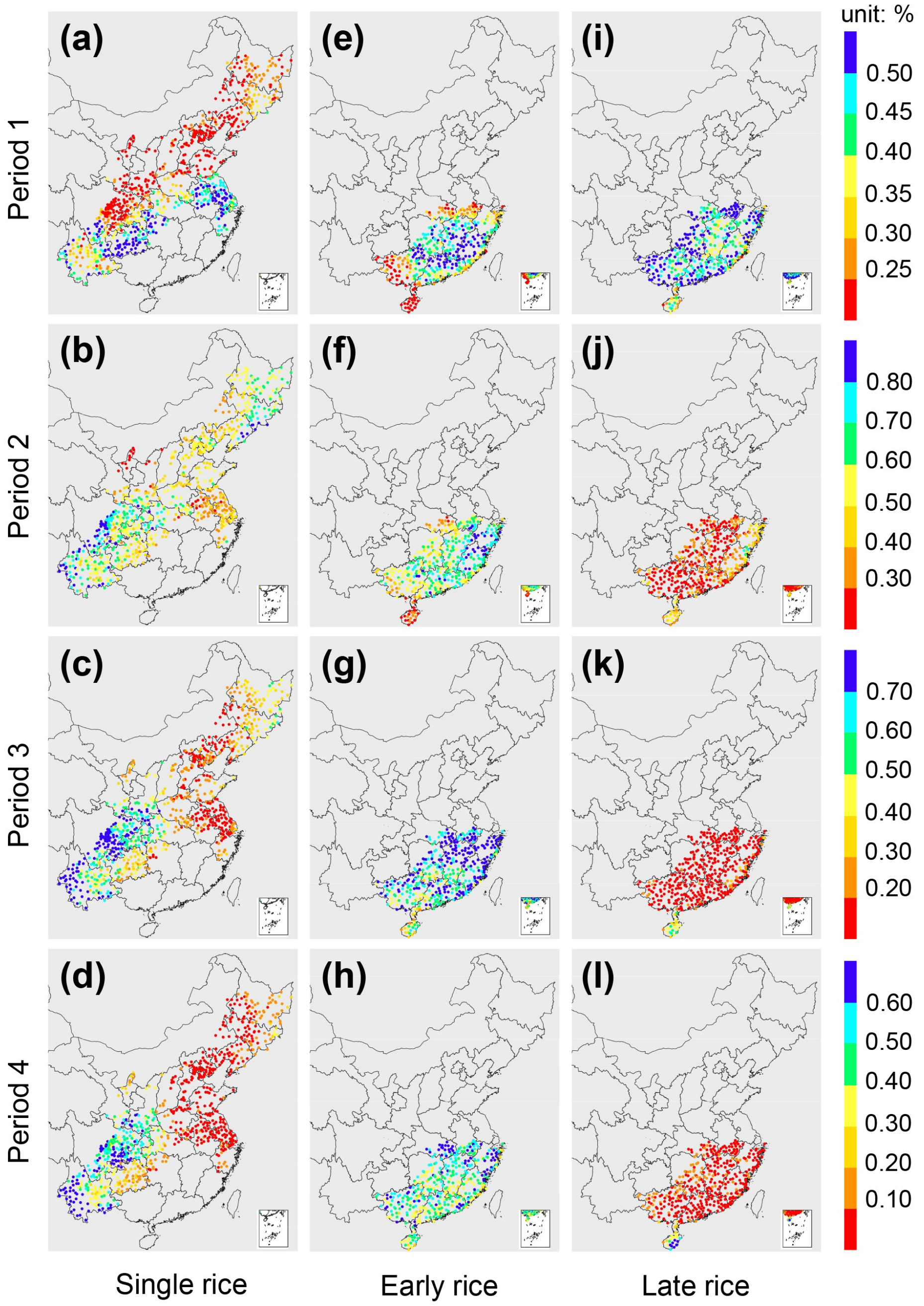

Figure S3. Spatial distribution of the frequency (%) of extreme precipitation averaged over the period 1961-2012 for four growing seasons (period 1: from transplanting to tillering; period 2: from the end of tillering to the end of flowering; period 3: from the end of flowering to doughty; and period 4: from maturity to harvesting) of single rice (left), early rice (middle) and late rice (right). Maps were generated in R version 3.6.0 (www.r-project.org)[1].

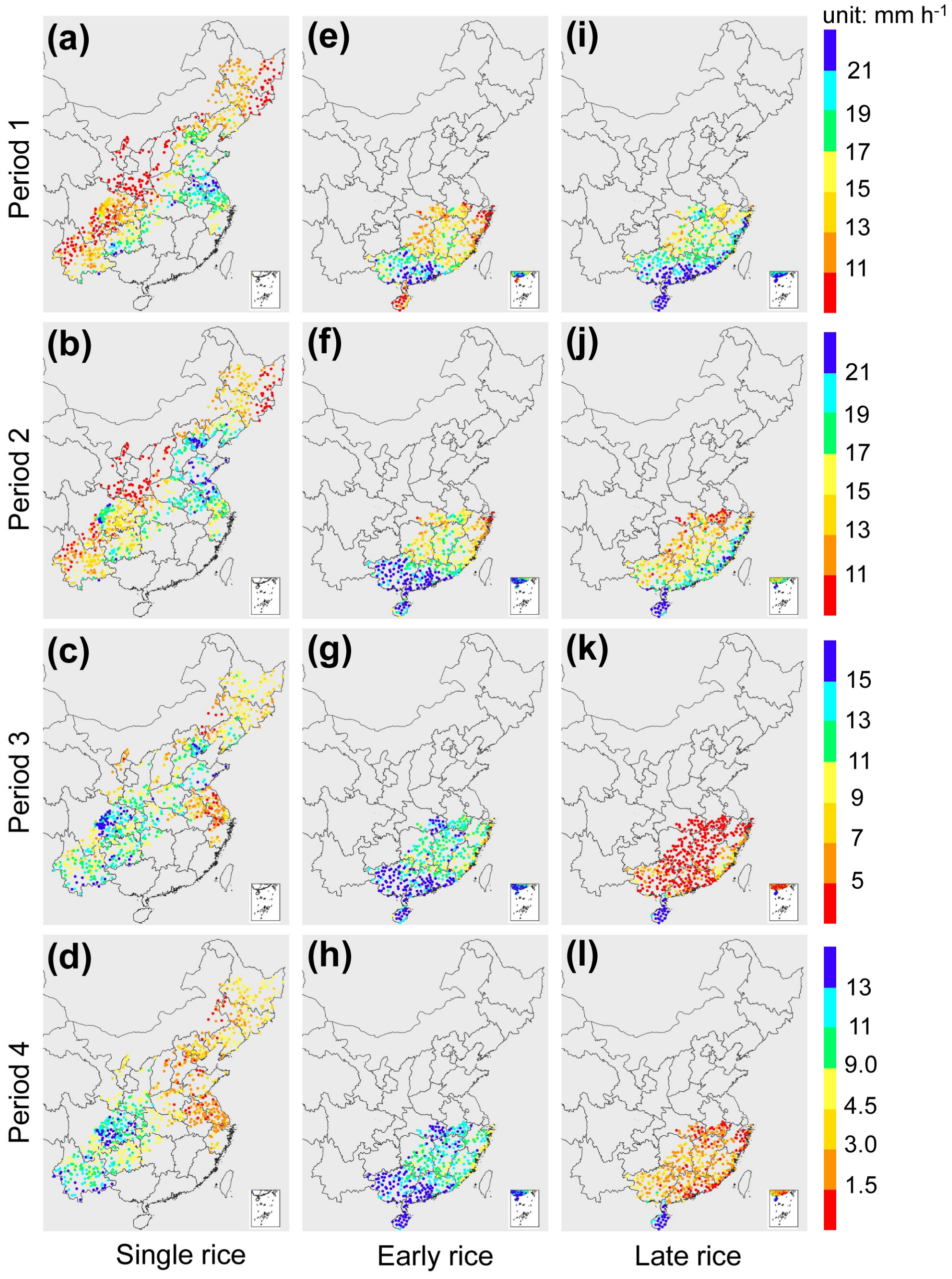

Figure S4. Spatial distribution of the intensity (mm h^{-1}) of extreme precipitation averaged over the period 1961-2012 for four growing seasons (period 1: from transplanting to tillering; period 2: from the end of tillering to the end of flowering; period 3: from the end of flowering to doughty; and period 4: from maturity to harvesting) of single rice (left), early rice (middle) and late rice (right). Maps were generated in R version 3.6.0 (www.r-project.org)[1].

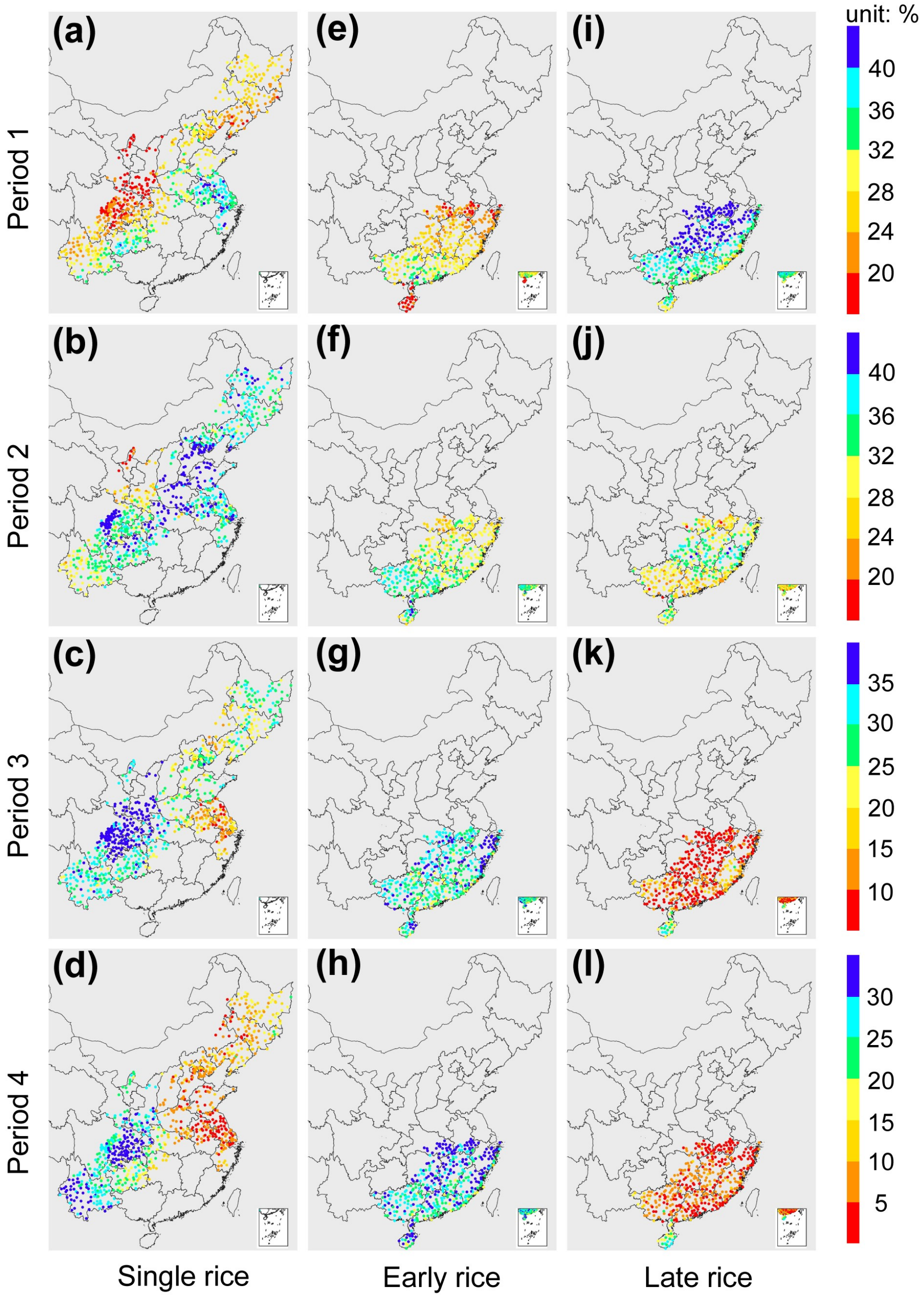

Figure S5. Spatial distribution of the proportion (%) of extreme precipitation averaged over the period 1961-2012 for four growing seasons (period 1: from transplanting to tillering; period 2: from the end of tillering to the end of flowering; period 3: from the end of flowering to doughty; and period 4: from maturity to harvesting) of single rice (left), early rice (middle) and late rice (right). Maps were generated in R version 3.6.0 (www.r-project.org)[1].

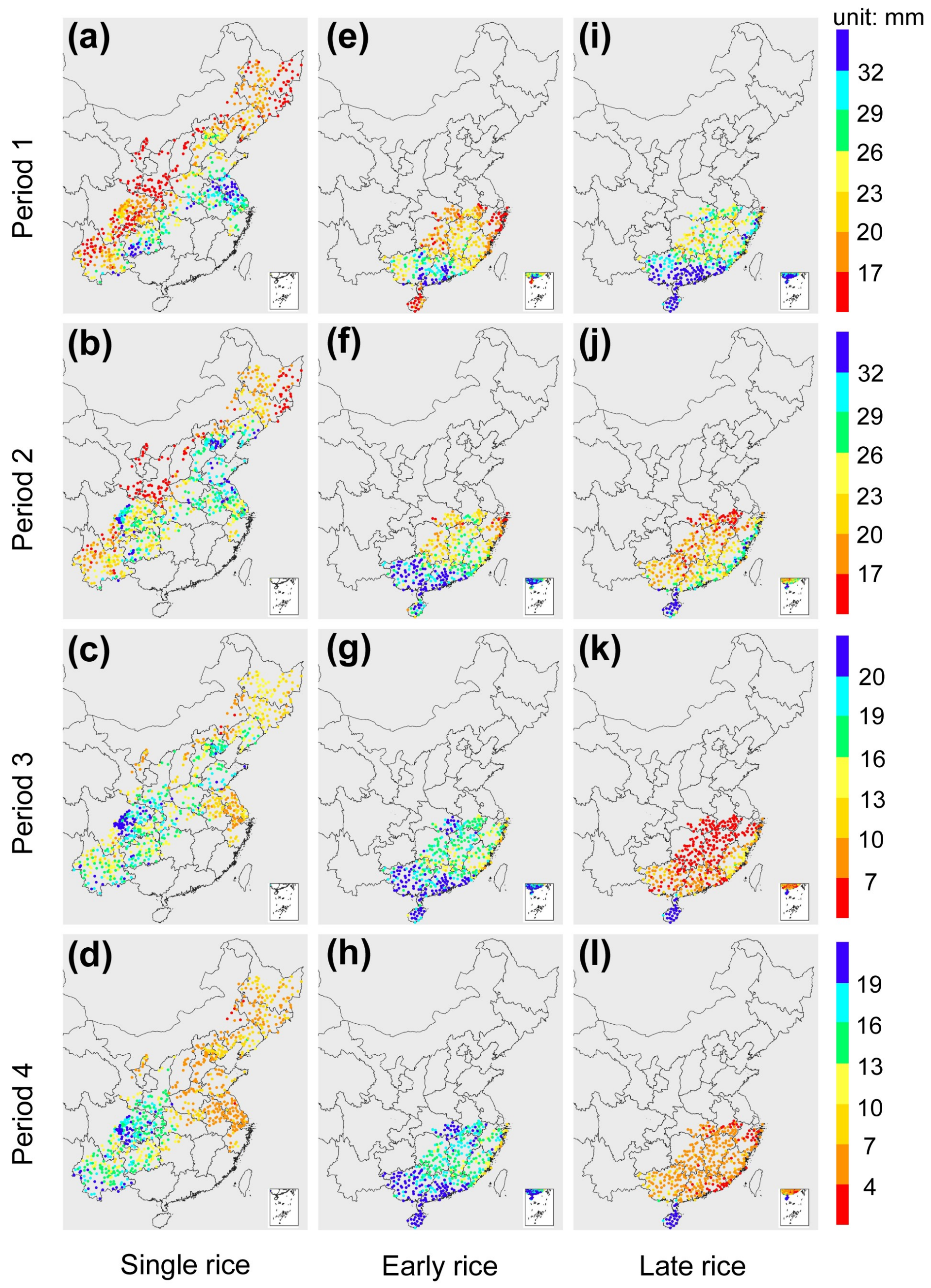

Figure S6. Spatial distribution of maximum hourly precipitation (mm) averaged over the period 1961-2012 for four growing seasons (period 1: from transplanting to tillering; period 2: from the end of tillering to the end of flowering; period 3: from the end of flowering to doughty; and period 4: from maturity to harvesting) of single rice (left), early rice (middle) and late rice (right). Maps were generated in R version 3.6.0 (www.r-project.org)[1].

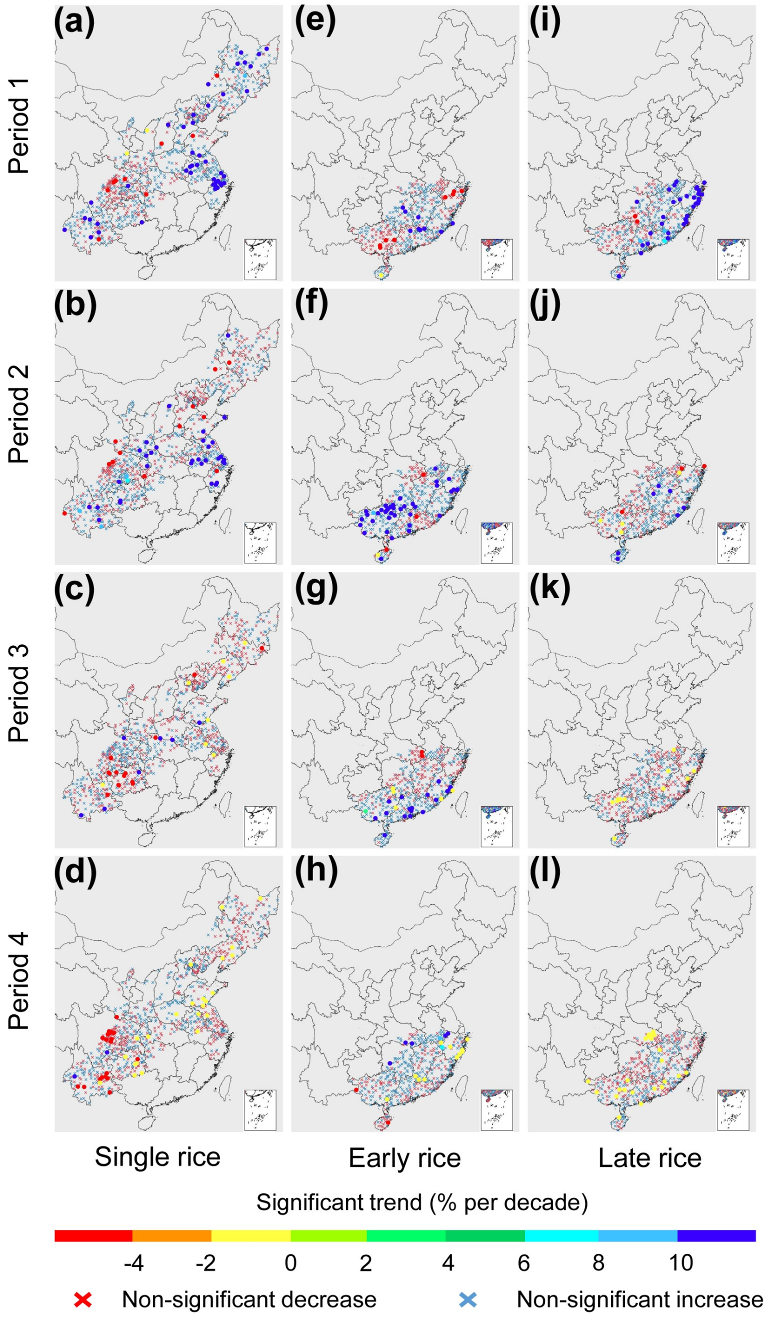

Figure S7. Spatial distribution of the temporal trends of the frequency of extreme precipitation during the period 1961-2012 for four growing seasons (period 1: from transplanting to tillering; period 2: from the end of tillering to the end of flowering; period 3: from the end of flowering to doughty; and period 4: from maturity to harvesting) of single rice (left), early rice (middle) and late rice (right). Maps were generated in R version 3.6.0 (www.r-project.org)[1].

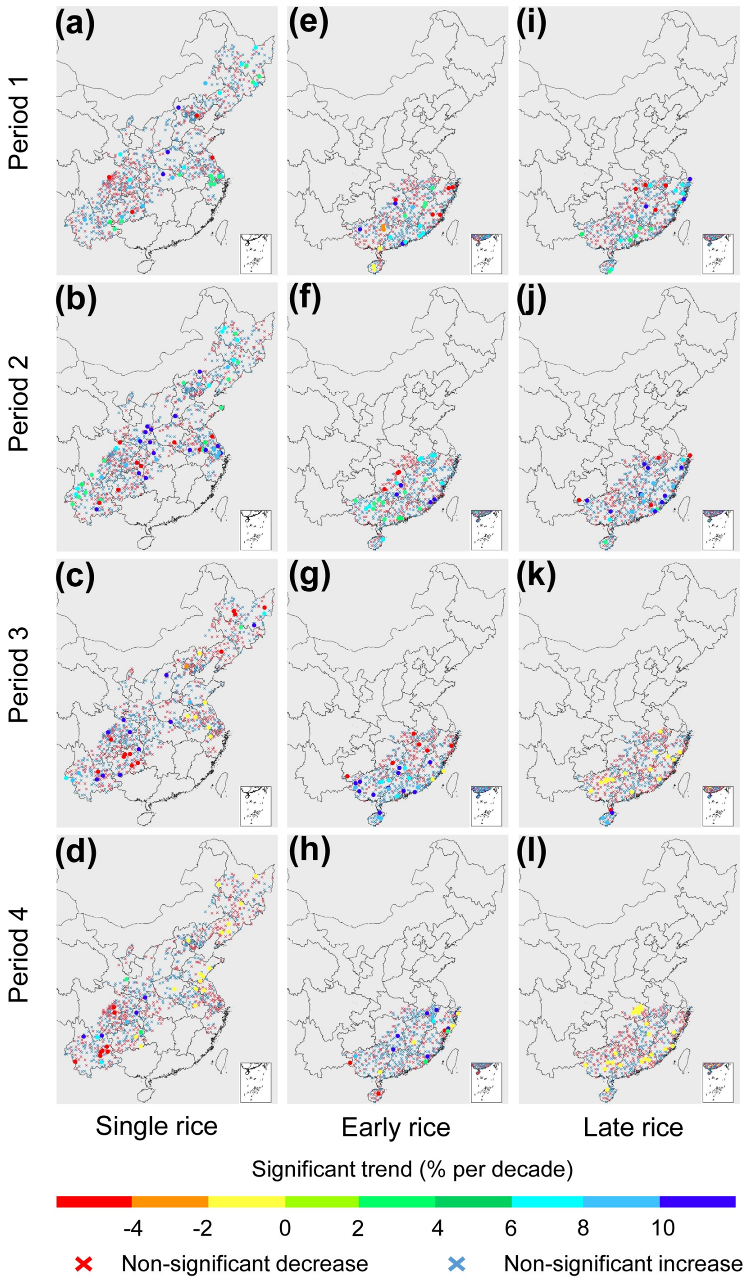

Figure S8. Spatial distribution of the temporal trends of the intensity of extreme precipitation during the period 1961-2012 for four growing seasons (period 1: from transplanting to tillering; period 2: from the end of tillering to the end of flowering; period 3: from the end of flowering to doughty; and period 4: from maturity to harvesting) of single rice (left), early rice (middle) and late rice (right)**.** Maps were generated in R version 3.6.0 (www.r-project.org)[1].

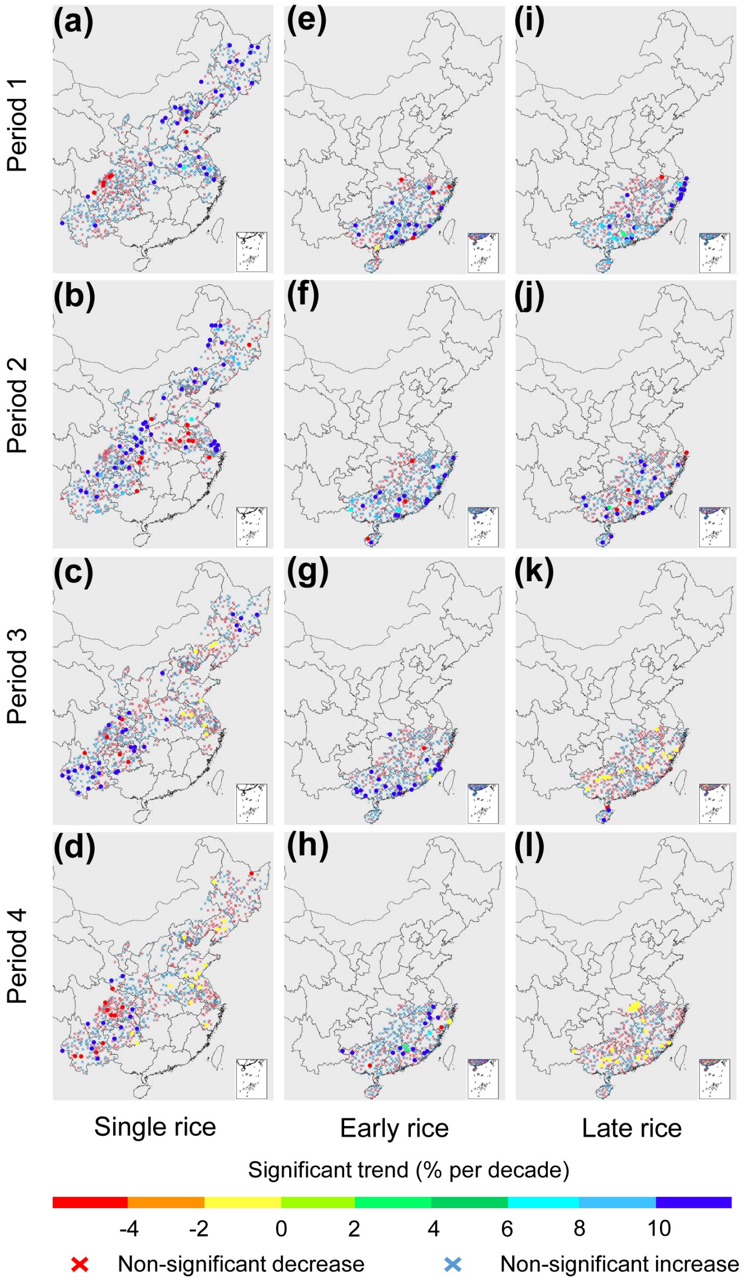

Figure S9. Spatial distribution of the temporal trends of the proportion of extreme precipitation during the period 1961-2012 for four growing seasons (period 1: from transplanting to tillering; period 2: from the end of tillering to the end of flowering; period 3: from the end of flowering to doughty; and period 4: from maturity to harvesting) of single rice (left), early rice (middle) and late rice (right). Maps were generated in R version 3.6.0 (www.r-project.org)[1].

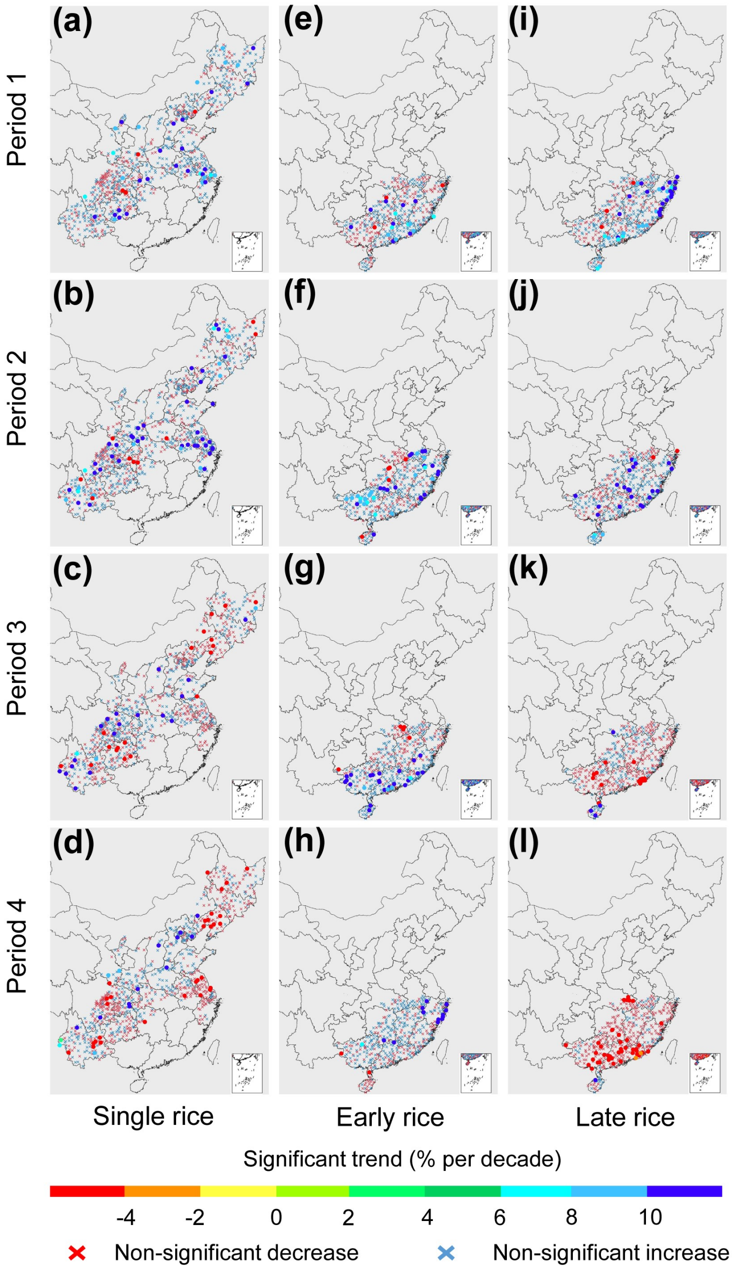

Figure S10. Spatial distribution of the temporal trends of maximum hourly precipitation during the period 1961-2012 for four growing seasons (period 1: from transplanting to tillering; period 2: from the end of tillering to the end of flowering; period 3: from the end of flowering to doughty; and period 4: from maturity to harvesting) of single rice (left), early rice (middle) and late rice (right). Maps were generated in R version 3.6.0 (www.r-project.org)[1].

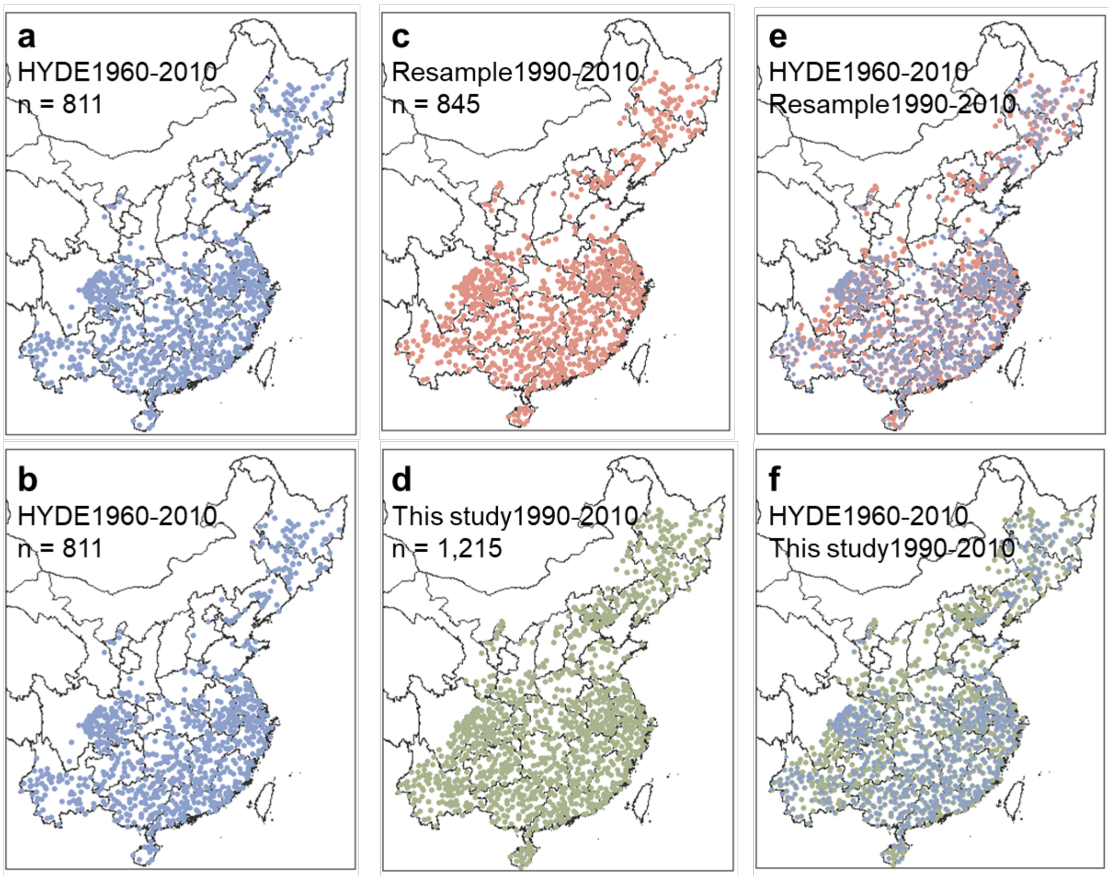

Figure S11. Meteorological stations of hourly precipitation datasets filtered by three union sets of rice paddy layers: (a, b) the union set at 5-arc-minute resolution from the History Database of the Global Environment (HYDE 3.2.1) from 1960 to 2010; (c) the union set at 5-arc-minute resolution resampled from high-resolution RS-derived maps in 1990-2010; (d) the RS-derived rice paddy maps at the spatial resolution of 1×1 km from the Chinese Academy of Sciences; (e) the overlapping layer of (a) and (c); (f) the overlapping layer of (b) and (d). Maps were generated in R version 3.6.0 (www.r-project.org)[1].

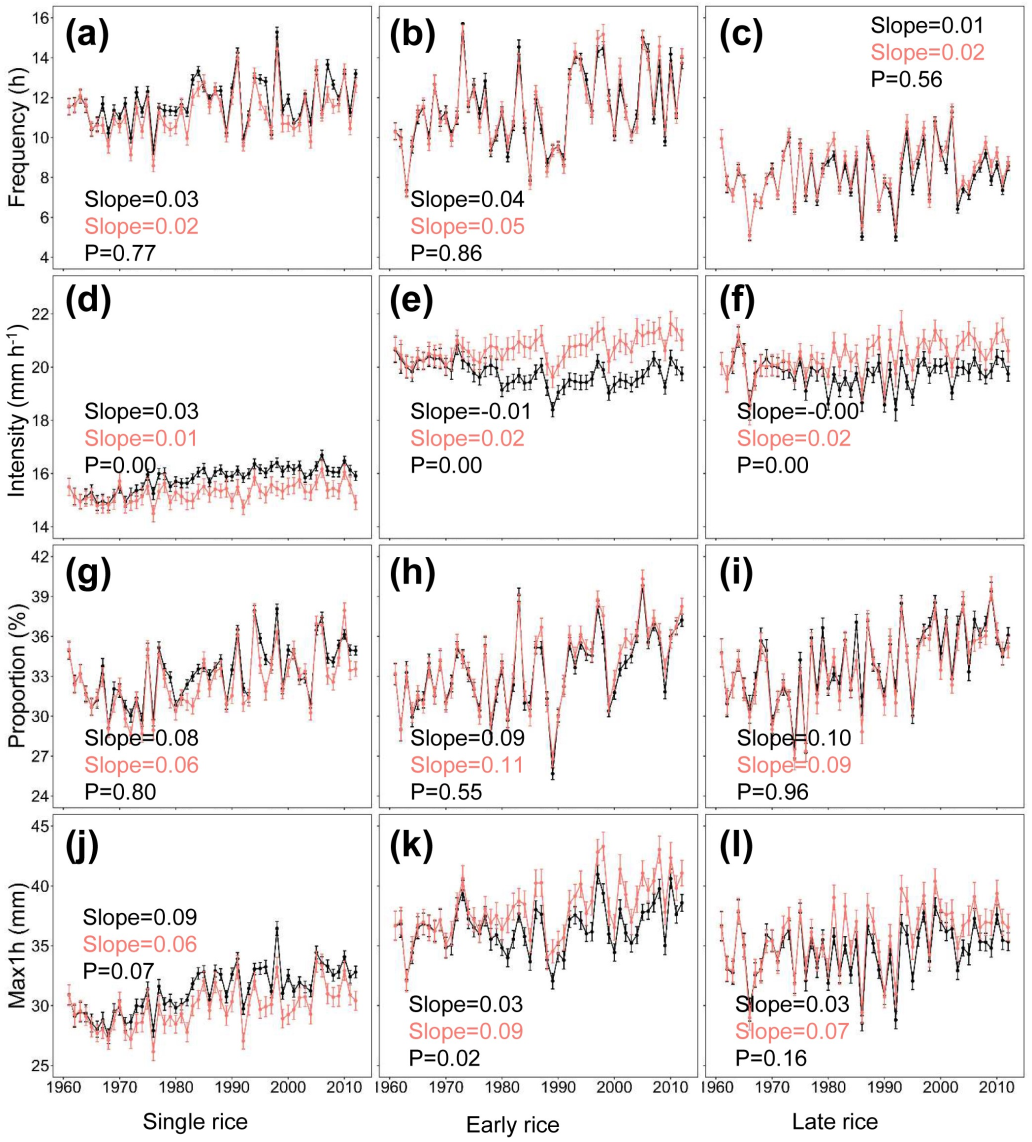

Figure S12. Sensitivity analysis. Black lines represent the means of all the available stations (813 stations for single rice, 412 stations for early/late rice). Red lines represent the means of all the stations without missing values (294 for single rice, 220 for early/late rice). Significance was tested between slopes of the two lines with $\alpha = 0.05$. Figures were generated in R version 3.6.0 (www.r-project.org)[1].

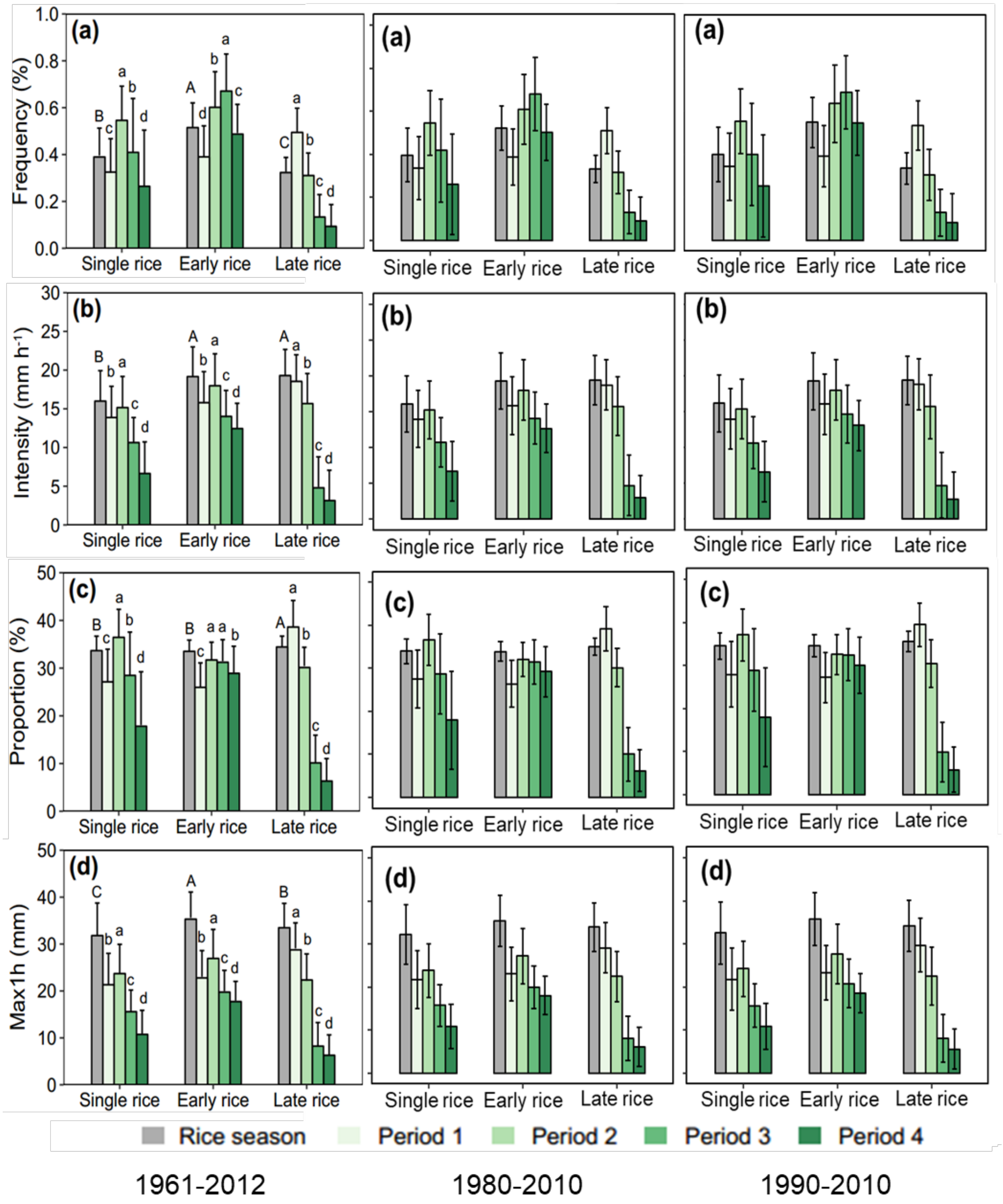

Figure. S13. Extreme precipitation indices by rice type and growing period during different study period. (left) 1961-2012; (middle) 1980-2010; (right) 1990-2010. Error bar indicates one standard deviation of extreme precipitation indices due to spatial variation. Figures were generated in R version 3.6.0 (www.r-project.org)[1].

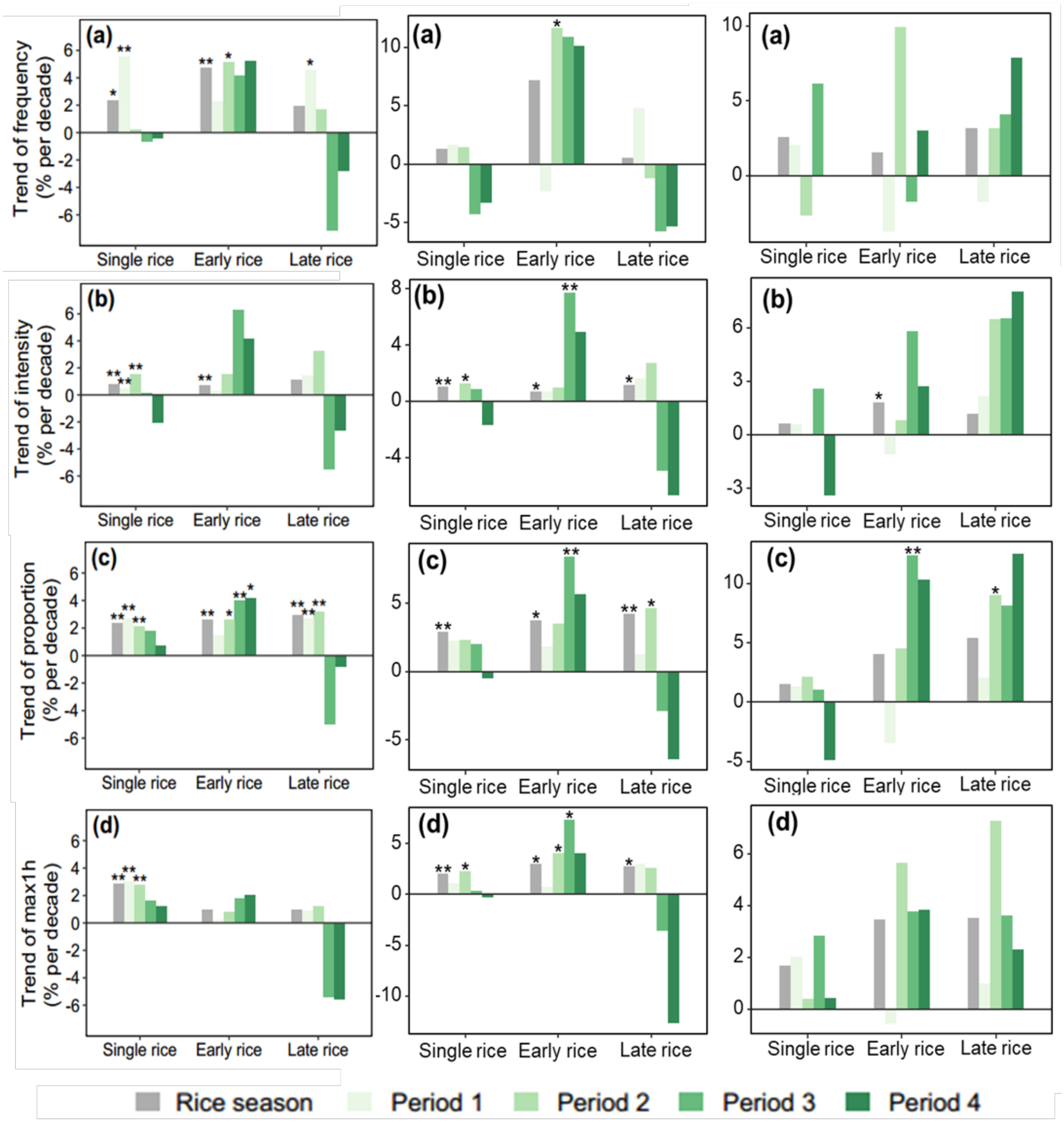

1961-2012 1980-2010 1990-2010

Figure. S14. Temporal trend of extreme precipitation indices by rice type and growing period during different study period. (left) 1961-2012; (middle) 1980-2010; (right) 1990-2010, with * p <0.05 and ** p <0.01. Figures were generated in R version 3.6.0 (www.r-project.org)[1].

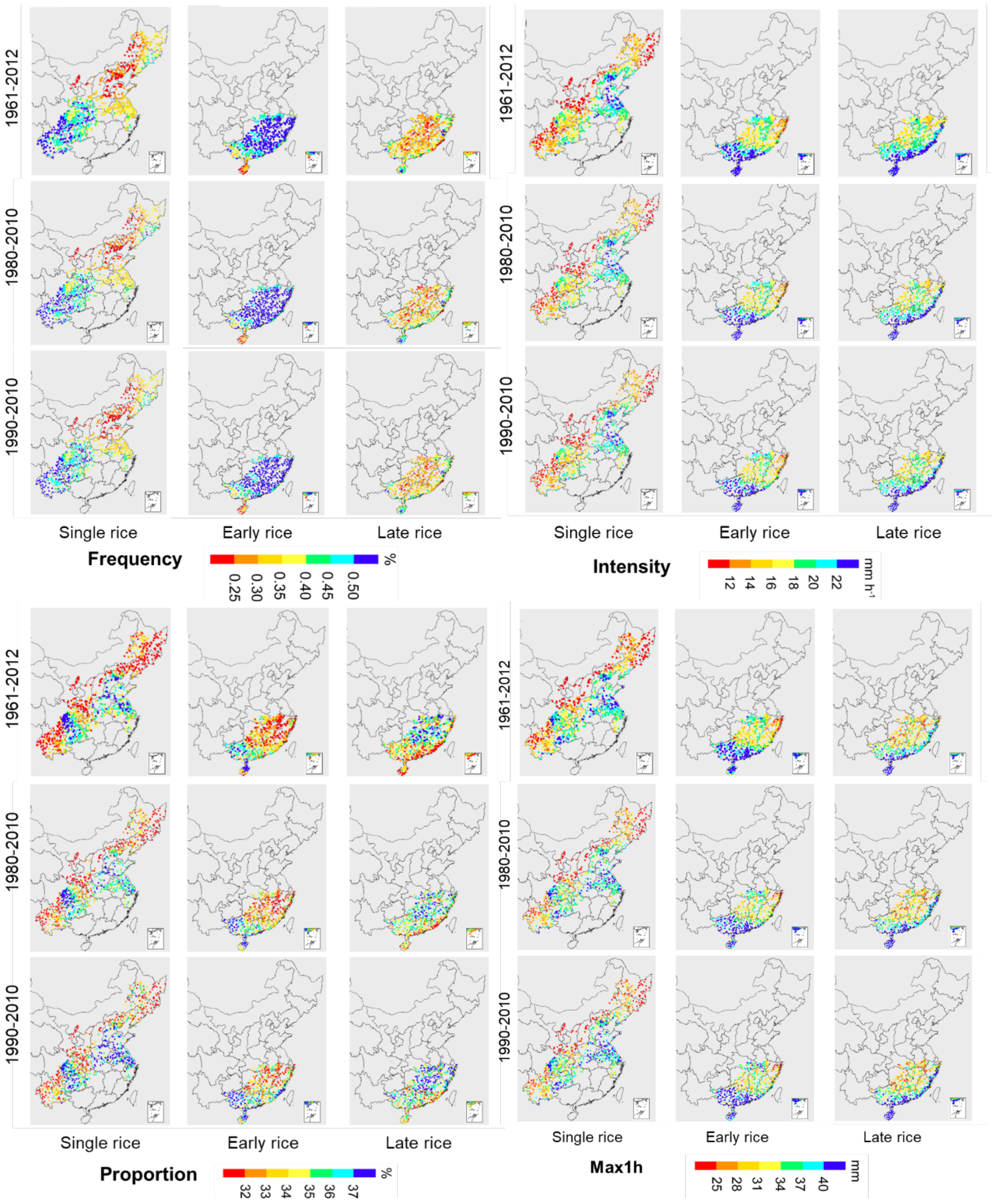

Figure. S15. Spatial distribution of extreme precipitation indices averaged over different study period (1961-2012, 1980-2010 and 1990-2010). Maps were generated in R version 3.6.0 (www.r-project.org)[1].

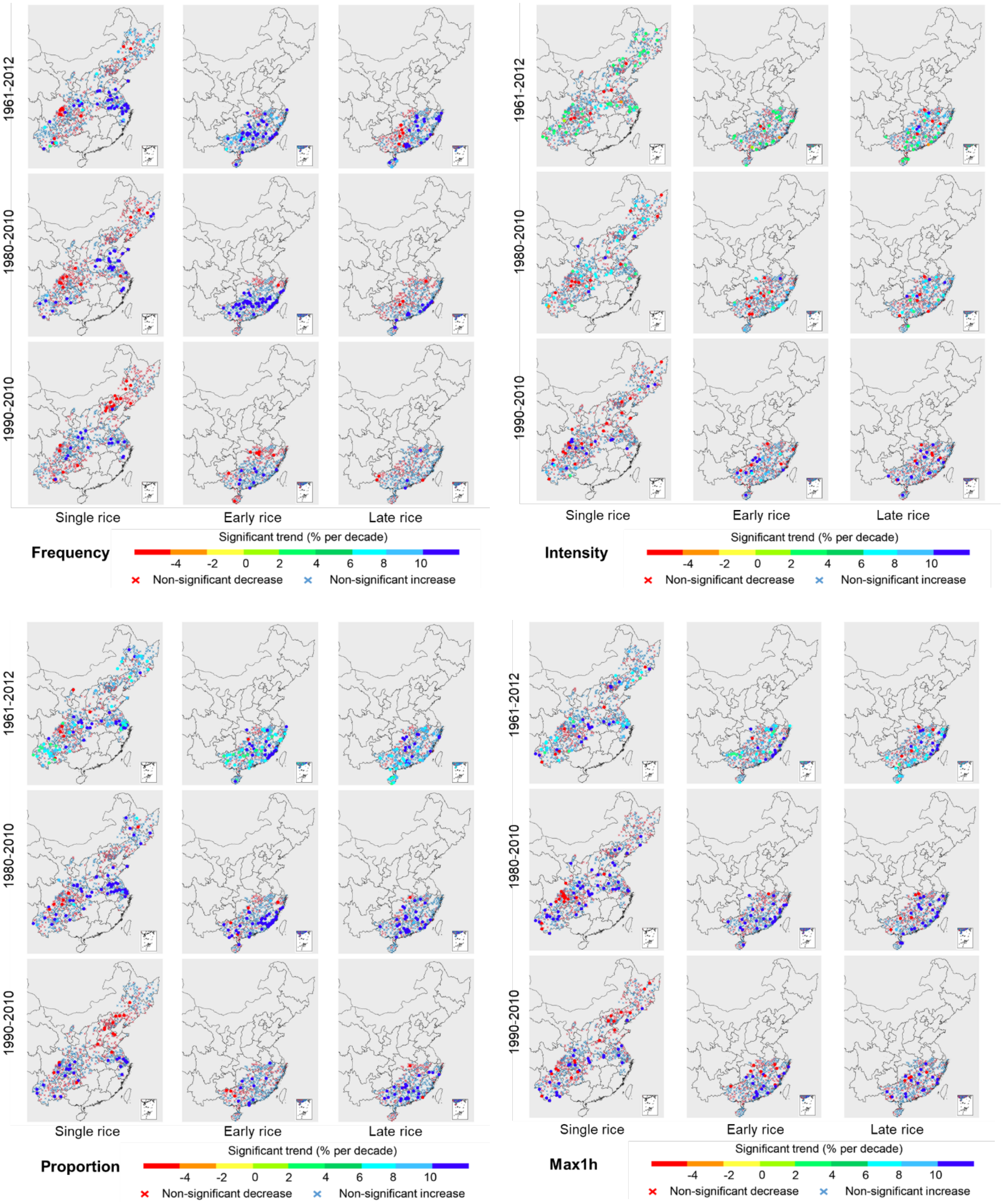

Figure. S16. Spatial distribution of the temporal trends of extreme precipitation indices during different study period (1961-2012, 1980-2010 and 1990-2010). Red crosses indicated for insignificant negative trends, blue crosses indicated for insignificant positive trends, while dots indicated for significant trends ($p<0.05$). Maps were generated in R version 3.6.0 (www.r-project.org)[1].

Table S1. Definition of extreme precipitation indices at the hourly scale.

Index	Definition	Unit	Time scale
Frequency	Ratio of the number of hours when hourly precipitation exceeded the 95th percentile threshold to the length of the growth period in hours	%	Entire rice season/each growing period
Intensity	Ratio of the amount of extreme precipitation and the frequency of extreme precipitation	mm h^{-1}	Entire rice season/each growing period
Proportion	Contribution of extreme precipitation to the total amount: the amount of extreme precipitation / total precipitation amount*100%	%	Entire rice season/each growing period
Max1h	Maximum hourly precipitation	mm	Entire rice season/each growing period

Table S2. Phenological information of rice cropping regions. Date is shown as month/day.

Rice cropping region	Sub-region	Cropping system	Length of rice growing period (day)	**Period 1** (from transplanting to tillering)		**Period 2** (from the end of tillering to the end of flowering)		**Period 3** (from the end of flowering to doughty)		**Period 4** (from maturity to harvesting)	
Northeast	Region 1	Single rice	134	5/15	7/15	7/16	8/15	8/16	9/5	9/6	9/25
	Region 2	Single rice	134	5/15	7/15	7/16	8/10	8/11	9/2	9/3	9/25
	Region 3	Single rice	129	5/20	7/15	7/16	8/10	8/11	8/31	9/1	9/25
	Region 4	Single rice	129	5/20	7/15	7/16	8/15	8/16	9/5	9/6	9/25
	Region 5	Single rice	129	5/20	7/15	7/16	8/15	8/16	9/5	9/6	9/25
	Region 6	Single rice	129	5/20	7/15	7/16	8/15	8/16	9/5	9/6	9/25
	Region 7	Single rice	134	5/20	7/15	7/16	8/20	8/21	9/10	9/11	9/30
	Region 8	Single rice	134	5/20	7/15	7/16	8/20	8/21	9/10	9/11	9/30
	Region 9	Single rice	134	5/20	7/15	7/16	8/20	8/21	9/10	9/11	9/30
	Region 10	Single rice	134	5/20	7/15	7/16	8/20	8/21	9/10	9/11	9/30
Huang-Huai-Hai Plain	Region 11	Single rice	134	5/20	7/15	7/16	8/15	8/16	9/9	9/10	9/30
	Region 12	Single rice	133	5/1	6/15	6/16	7/10	7/11	8/10	8/11	9/10
Middle and lower Yangtze River	Region 13	Single rice	133	6/10	8/5	8/6	9/10	9/11	9/30	10/1	10/20
	Region 14	Single rice	133	6/10	7/31	8/1	9/10	9/11	9/30	10/1	10/20
	Region 15	Single rice	123	6/15	7/30	7/31	9/5	9/6	9/25	9/26	10/15
	Region 16	Single rice	108	6/25	8/6	8/7	9/10	9/11	9/25	9/26	10/10
	Region 17	Single rice	113	6/25	8/11	8/12	9/15	9/16	9/30	10/1	10/15
	Region 18	Single rice	118	6/10	7/20	7/21	9/1	9/2	9/20	9/21	10/5
	Region 19	Single rice	118	6/10	7/25	7/26	9/1	9/2	9/20	9/21	10/5
	Region 20	Single rice	114	5/30	7/10	7/11	8/10	8/11	8/30	8/31	9/20

	Region 21	Single rice	108	6/5	7/20	7/21	8/21	8/22	9/5	9/6	9/20
	Region 22	Single rice	114	5/20	7/10	7/11	8/11	8/12	8/26	8/27	9/10
Southwest	Region 23	Single rice	123	4/5	5/25	5/26	7/6	7/7	7/21	7/22	8/6
	Region 24	Single rice	123	4/15	6/15	6/16	7/12	7/13	7/31	8/1	8/15
	Region 25	Single rice	124	5/10	7/5	7/6	8/5	8/6	8/22	8/23	9/10
	Region 26	Single rice	123	4/15	6/1	6/2	7/16	7/17	7/31	8/1	8/15
	Region 27	Single rice	128	4/5	5/25	5/26	7/15	7/16	7/28	7/29	8/10
	Region 28	Single rice	134	5/15	6/30	7/1	8/15	8/16	9/5	9/6	9/25
	Region 29	Single rice	129	5/15	6/30	7/1	8/10	8/11	8/30	8/31	9/20
	Region 30	Single rice	133	4/15	6/15	6/16	7/20	7/21	8/7	8/8	8/25
	Region 31	Single rice	124	5/15	6/30	7/1	8/10	8/11	8/28	8/29	9/15
Southern China	Region 32	Early rice	98	3/10	4/11	4/12	5/16	5/17	5/31	6/1	6/15
		Late rice	107	6/16	7/27	7/28	8/31	9/1	9/15	9/16	9/30
	Region 33	Early rice	106	4/1	5/11	5/12	6/15	6/16	6/30	7/1	7/15
		Late rice	113	7/16	9/5	9/6	10/10	10/11	10/23	10/24	11/5
	Region 34	Early rice	101	4/1	5/10	5/11	6/15	6/16	6/27	6/28	7/10
		Late rice	114	7/11	8/25	8/26	10/2	10/3	10/17	10/18	11/1
	Region 35	Early rice	92	4/15	5/20	5/21	6/20	6/21	6/30	7/1	7/15
		Late rice	97	7/16	8/16	8/17	9/20	9/21	10/5	10/6	10/20
Middle and lower Yangtze River	Region 36	Early rice	87	4/25	5/25	5/26	6/20	6/21	7/5	7/6	7/20
		Late rice	102	7/21	8/22	8/23	9/30	10/1	10/15	10/16	10/30
	Region 37	Early rice	87	4/20	5/20	5/21	6/15	6/16	6/30	7/1	7/15
		Late rice	102	7/16	8/15	8/16	9/20	9/21	10/8	10/9	10/25
	Region38	Early rice	87	4/20	5/20	5/21	6/20	6/21	7/3	7/4	7/15
		Late rice	102	7/16	8/20	8/21	9/25	9/26	10/10	10/11	10/25
	Region 39	Early rice	92	4/15	5/20	5/21	6/20	6/21	6/30	7/1	7/15

	Late rice	102	7/16	8/20	8/21	9/25	9/26	10/10	10/11	10/25
Region 40	Early rice	87	4/20	5/20	5/21	6/20	6/21	6/30	7/1	7/15
	Late rice	102	7/16	8/20	8/21	9/25	9/26	10/10	10/11	10/25
Region 41	Early rice	87	4/25	5/25	5/26	6/20	6/21	7/5	7/6	7/20
	Late rice	92	7/21	8/20	8/21	9/20	9/21	10/5	10/6	10/20
Region 42	Early rice	92	4/25	5/30	5/31	6/30	7/1	7/12	7/13	7/25
	Late rice	99	7/26	8/30	8/31	9/30	10/1	10/16	10/17	11/1
Region 43	Early rice	87	4/25	5/30	5/31	6/25	6/26	7/8	7/9	7/20
	Late rice	102	7/21	8/26	8/27	9/30	10/1	10/15	10/16	10/30
Region44	Early rice	82	4/30	5/25	5/26	6/20	6/21	7/5	7/6	7/20
	Late rice	87	7/21	8/20	8/21	9/20	9/21	10/2	10/3	10/15
Region 45	Early rice	82	4/30	5/25	5/26	6/20	6/21	7/5	7/6	7/20
	Late rice	87	7/21	8/20	8/21	9/15	9/16	9/30	10/1	10/15

Table S3 Correlation coefficients of rice yield and extreme precipitation indices (1981-2012) at provincial scale for single rice during four rice growing periods. We denote those significant at the 10% level with an asterisk, those significant at the 5% level with two asterisks.

Province	Frequency				Intensity				Proportion				Max1h			
	Period 1	Period 2	Period 3	Period 4	Period 1	Period 2	Period 3	Period 4	Period 1	Period 2	Period 3	Period 4	Period 1	Period 2	Period 3	Period 4
Anhui	0.00	0.03	-0.33*	-0.17	0.06	0.26	-0.37**	-0.12	-0.12	0.12	-0.46**	-0.01	0.08	0.34*	-0.28	-0.27
Beijing	0.40**	0.18	-0.04	-0.01	-0.15	0.24	0.15	-0.02	0.21	0.08	0.19	-0.16	0.09	0.28	0.09	-0.03
Gansu	-0.20	-0.09	-0.10	-0.20	0.16	-0.16	0.16	-0.10	-0.10	-0.09	0.24	-0.08	0.19	-0.08	-0.11	-0.04
Guizhou	0.06	0.45**	0.23	0.09	0.06	0.14	0.51**	0.06	0.01	0.16	0.58**	0.00	0.00	0.23	0.40**	0.02
Hebei	0.28	0.10	0.03	-0.11	0.37**	0.07	0.16	-0.07	0.31*	0.42**	-0.10	-0.08	0.29	0.12	0.16	-0.12
Henan	0.08	0.18	-0.09	0.34*	0.13	0.17	0.00	0.31*	0.11	-0.20	0.21	0.34*	-0.02	0.11	0.00	0.46**
Heilongjiang	-0.01	-0.26	0.14	0.18	0.11	0.05	0.17	0.14	-0.03	0.11	0.15	0.10	0.08	-0.04	0.16	0.23
Hubei	0.14	-0.17	0.04	-0.18	0.27	0.08	0.01	-0.20	0.25	0.03	-0.02	0.03	0.26	0.02	-0.01	-0.21
Hunan	0.02	-0.42**	0.03	-0.04	-0.16	0.21	0.02	0.01	-0.21	-0.10	0.05	-0.13	-0.14	0.03	0.02	-0.03
Jilin	-0.05	-0.06	0.05	-0.06	-0.28	-0.12	0.16	0.04	-0.18	0.01	0.19	0.03	-0.17	-0.05	0.20	0.11
Jiangsu	0.13	-0.22	-0.13	-0.31*	0.24	-0.30*	-0.21	-0.24	0.26	-0.33*	-0.25	-0.19	0.25	-0.20	-0.14	-0.28
Liaoning	-0.08	-0.29	0.19	0.23	-0.22	0.32*	0.18	0.27	-0.24	0.13	0.25	0.23	-0.05	-0.02	0.18	0.23
Inner Mongolia	-0.21	0.07	-0.22	-0.43**	-0.07	0.06	-0.05	-0.36**	-0.20	0.14	0.06	-0.27	-0.01	0.13	-0.12	-0.22
Ningxia	0.18	-0.11	-0.36**	-0.22	0.40**	0.00	-0.08	-0.22	0.19	0.00	-0.04	-0.14	0.33*	-0.07	-0.12	-0.27
Shandong	0.10	0.36**	-0.16	0.27	0.31*	0.09	-0.02	0.24	0.26	0.37**	-0.03	0.24	-0.06	0.17	-0.02	0.07
Shanxi	0.02	-0.25	0.28	-0.37**	0.05	-0.01	0.08	-0.27	-0.05	-0.15	0.16	-0.31*	-0.02	-0.25	-0.06	-0.28
Shaanxi	0.28	0.21	0.03	-0.14	0.09	0.27	0.08	-0.17	0.12	0.21	-0.17	-0.31*	0.12	0.26	0.12	-0.08
Shanghai	-0.01	-0.14	-0.58**	-0.35*	0.11	0.10	-0.59**	-0.37**	-0.14	0.01	-0.62**	-0.29	0.16	0.11	-0.63**	-0.42**
Sichuan	0.04	0.06	-0.01	0.20	0.21	-0.16	0.20	0.30*	-0.04	-0.29	0.00	0.31*	0.21	-0.06	0.09	0.30
Tianjin	0.40**	0.29	0.01	0.10	0.03	0.14	0.23	0.10	0.16	0.28	0.19	0.12	0.21	0.13	0.07	-0.16
Yunnan	0.21	-0.07	-0.40**	-0.26	0.15	0.00	0.11	0.13	-0.10	0.38**	0.18	-0.14	0.18	0.16	-0.11	-0.10
Zhejiang	0.22	-0.17	-0.06	0.06	0.08	0.16	-0.01	0.08	0.11	0.06	-0.05	0.14	0.25	0.12	0.00	0.08
Chongqing	-0.22	0.17	-0.11	-0.11	-0.29	0.27	-0.03	-0.03	-0.29	0.26	-0.04	0.00	-0.35*	0.23	-0.09	0.01

Table S4 Correlation coefficients of rice yield and extreme precipitation indices (1981-2012) at provincial scale for early rice during four rice growing periods. We denote those significant at the 10% level with an asterisk, those significant at the 5% level with two asterisks.

Province	Frequency				Intensity				Proportion				Max1h			
	Period 1	Period 2	Period 3	Period 4	Period 1	Period 2	Period 3	Period 4	Period 1	Period 2	Period 3	Period 4	Period 1	Period 2	Period 3	Period 4
Anhui	-0.30	-0.30	-0.64**	-0.03	0.02	0.12	-0.41**	0.16	-0.05	0.02	-0.28	0.18	-0.07	-0.08	-0.46**	0.15
Fujian	0.01	-0.30	-0.05	0.22	0.01	-0.15	0.35*	0.20	-0.22	0.17	0.25	0.20	-0.03	-0.24	0.24	0.22
Guangdong	-0.33*	-0.51**	-0.17	-0.19	-0.25	-0.07	-0.01	-0.05	-0.40**	-0.04	0.16	0.08	-0.45**	-0.35*	-0.12	-0.16
Guangxi	0.11	-0.29	-0.50**	-0.10	0.37**	-0.31*	-0.43**	-0.15	0.27	0.00	-0.07	0.12	0.26	-0.27	-0.42**	-0.14
Hainan	0.52**	-0.11	0.06	0.17	0.46**	-0.12	-0.09	0.09	0.56**	-0.16	-0.02	-0.06	0.49**	0.02	0.04	0.12
Hubei	-0.36**	-0.02	-0.26	-0.12	-0.28	0.01	-0.07	-0.27	-0.20	0.01	0.01	-0.24	-0.27	0.02	-0.11	-0.18
Hunan	-0.09	0.11	-0.17	-0.06	-0.10	0.10	-0.04	0.32*	0.02	0.12	-0.02	0.17	-0.10	0.23	-0.02	0.25
Jiangxi	-0.32*	-0.19	-0.72**	-0.02	-0.20	-0.20	-0.31*	0.07	-0.25	-0.07	-0.17	0.03	-0.18	-0.39**	-0.42**	0.03
Zhejiang	-0.44**	0.09	-0.26	-0.01	-0.39**	0.22	-0.03	-0.18	-0.16	0.17	0.11	0.16	-0.32*	0.27	0.00	-0.03

Table S5 Correlation coefficients of rice yield and extreme precipitation indices (1981-2012) at provincial scale for late rice during four growing periods. We denote those significant at the 10% level with an asterisk, those significant at the 5% level with two asterisks.

Province	Frequency				Intensity				Proportion				Max1h			
	Period 1	Period 2	Period 3	Period 4	Period 1	Period 2	Period 3	Period 4	Period 1	Period 2	Period 3	Period 4	Period 1	Period 2	Period 3	Period 4
Anhui	0.21	-0.16	-0.16	0.15	-0.17	-0.27	-0.22	0.18	0.08	-0.26	-0.09	0.17	-0.05	-0.18	-0.21	0.20
Fujian	0.04	-0.07	-0.01	-0.03	0.02	0.10	0.04	0.07	0.04	0.13	0.01	0.05	0.03	0.05	0.05	0.09
Guangdong	-0.14	-0.18	-0.10	-0.10	-0.33*	0.01	-0.07	-0.08	-0.37**	-0.27	0.01	-0.09	-0.26	-0.15	-0.07	-0.16
Guangxi	-0.27	-0.09	-0.02	0.17	-0.20	0.20	-0.02	0.17	-0.16	0.19	0.05	0.24	-0.32*	0.14	-0.07	0.07
Hainan	0.50**	-0.02	-0.30*	-0.26	-0.31*	0.08	-0.21	-0.28	0.09	0.03	-0.22	-0.37**	-0.10	-0.03	-0.16	-0.26
Hubei	-0.33*	0.24	-0.22	0.09	-0.18	0.12	-0.07	0.10	0.00	0.14	-0.05	0.09	-0.22	0.17	-0.17	-0.01
Hunan	0.23	-0.16	-0.08	-0.11	0.35*	0.02	-0.04	-0.16	0.20	0.34*	0.05	-0.11	0.30	-0.06	-0.01	-0.24
Jiangxi	0.00	0.10	-0.04	0.12	0.18	0.17	-0.04	0.11	-0.07	0.09	-0.03	0.13	0.07	0.11	0.02	0.13
Zhejiang	-0.08	-0.67**	-0.54**	-0.17	0.08	-0.01	-0.50**	-0.09	0.19	-0.45**	-0.41**	-0.18	0.05	-0.41**	-0.53**	-0.10

Table S6. Multiple linear regression results. *p<0.1; **p<0.05; ***p<0.01

Variable	Single rice		Early rice		Late rice	
	Standardized coefficient	95% CI	Standardized coefficient	95% CI	Standardized coefficient	95% CI
Precipitation	-0.08	(-0.18,0.03)	-0.52***	(-0.69,-0.34)	-0.34***	(-0.52,-0.16)
Temperature			-0.16*	(-0.29,-0.04)	0.18**	(0.05,0.31)
Solar radiation			0.21**	(0.05,0.36)		
Frequency_P1					0.39***	(0.20,0.57)
Frequency_P3	-0.08	(-0.18,0.03)	-0.49***	(-0.66,-0.32)		
Frequency_P4	-0.11	(-0.21,0.00)				
Intensity_P2	0.12**	(0.05,0.20)			0.31**	(0.12,0.51)
Intensity_P3					-0.48*	(-0.91,-0.05)
Intensity_P4			0.09	(-0.01,0.19)		
Proportion_P1	-0.11	(-0.23,0.00)	-0.17	(-0.35,0.01)		
Proportion_P2			-0.12	(-0.29,0.05)	-0.25**	(-0.43,-0.06)
Proportion_P3	0.13**	(0.03,0.22)				
Proportion_P4	-0.11	(-0.23,0.02)				
Max1h_P1	0.18**	(0.05,0.31)	0.36***	(0.16,0.55)	-0.16	(-0.32,0.00)
Max1h_P2			0.23*	(0.04,0.42)		
Max1h_P3			0.33***	(0.18,0.49)	0.50*	(0.05,0.95)
Max1h_P4	0.18*	(0.03,0.33)				
Adjusted R^2	0.03		0.41		0.11	

Table S7 Differences in the 95th percentile thresholds (*T*) compared to those of the period 1981-2010. Four candidate base periods were considered: 1976-2005, 1971-2000, 1971-2010, and 1961-2012. The difference is computed as the difference in *T* between candidate base period and 1981-2010 divided by *T* of the period 1981-2010.

Type	**1971-2000**	**1976-2005**	**1971-2010**	**1961-2012**
Single rice	-1.11±3.95	-0.90±2.69	-0.85±2.21	-0.88±2.66
Early rice	-2.29±3.90	-1.32±2.81	-0.65±2.03	-0.83±2.68
Late rice	-3.18±4.56	-1.71±3.32	-1.51±2.53	-1.74±3.34

References

1 Team. R. core.R: A language and environment for statistical computing. (2018).
